# Reflexology in oncological treatment – a systematic review

**DOI:** 10.1186/s12906-023-04220-4

**Published:** 2024-01-11

**Authors:** Moritz Klaus, Sabine Kutschan, Heidrun Männle, Jutta Hübner, Jennifer Dörfler

**Affiliations:** 1 Klinik Für Innere Medizin II, Hämatologie Und Internistische Onkologie, Universitätsklinikum Jena, Am Klinikum 1, 07747 Jena, Germany; 2 Gynäkologie Und Geburtshilfe, Ortenau-Klinikum Offenburg-Kehl, Ebertplatz 12, 77654 Offenburg, Germany

**Keywords:** Cancer, Reflexology, Reflex zones, Oncological treatment

## Abstract

**Background:**

As cancer and its therapy comes with a wide range of negative effects, people look for options to mitigate these effects. Reflexology is among the options of complementary medicine.

**Method:**

In March 2022 a systematic search was conducted searching five electronic databases (Embase, Cochrane, PsychInfo, CINAHL and Medline) to find studies concerning the use, effectiveness and potential harm of reflexology on cancer patients.

**Results:**

From all 821 search results, 29 publications concerning 26 studies with 2465 patients were included in this systematic review. The patients treated with reflexology were mainly diagnosed with breast, lung, gastrointestinal and hematological cancer. Outcomes were mainly pain, quality of life, anxiety, depression, fatigue. The studies had moderate to low quality and reported heterogeneous results: Some studies reported significant improvements in above mentioned outcomes while other studies did not find any changes concerning these endpoints.

**Conclusion:**

Due to the very heterogeneous results and methodical limitations of the included studies, a clear statement regarding the effectiveness of reflexology on cancer patients is not possible. The current evidence indicates that reflexology is superior to passive control groups for pain, quality of life and fatigue, however, more studies with comparable active control groups are needed.

**Supplementary Information:**

The online version contains supplementary material available at 10.1186/s12906-023-04220-4.

## Introduction

Cancer is a very prevalent disease with more than 18 million diagnosed cases worldwide in 2020 [1]. Due to the burden of the disease and adverse effects of cancer treatment, people look for options that might help mitigate these negative effects, with reflexology being a particularly popular option among complementary medicine. Reflexology involves applying manual pressure to specific parts of the body (often feet, sometimes hands) that are thought to correspond with specific internal organs. The stimulation of the body zones is intended to promote the self-healing powers of the organs that are associated with the respective zones. Originally developed as so called “Zone therapy” by William Fitzgerald, Eunice Ingham refined these techniques [2] and her method of reflexology is still used today. These reflex zones are also not to be confused with so called Head zones, named after neurologist Henry Head. He found that visceral diseases can result in hyperalgesia or allodynia of respective cutaneous areas [3].

This review aims at assessing clinical studies on the influence of reflexology as complementary medicine on cancer related symptoms and side effects of cancer therapy. It is not clear yet, whether differences in application might yield different results for a variety of outcomes in the context of cancer patients, which also applies to acute and long-term effects. Additionally, comparisons with other interventions that aim at improving the patients’ condition may help shed more light on the efficacy of reflexology. This exploration may help guide how healthcare practitioners can support cancer patients’ symptoms better and if reflexology can be an adequate tool in doing so.

## Method

### Criteria for including and excluding studies in the review

Inclusion and exclusion criteria are listed in Table 1 based on a PICO- model. Generally, all original studies with a randomized controlled design were included if they reported patient-relevant outcomes after treatment of adult cancer patients with any intervention containing reflexology. Because of the wide range of application fields, all cancer entities were included. Criteria for rejecting studies were primary prevention, grey literature, other publication type than primary investigation/report (e.g. comments, letters, abstracts) and study population with precancerous conditions. Additionally, studies were excluded if they reported no patient centered outcomes. Language restrictions were made to English and German. In order to shed more light on the effectiveness of reflexology compared to other non-specific interventions a distinction was made between active and passive control groups.

**Table 1:** PICO criteria

PICO	Inclusion criteria	Exclusion criteria
Patient	Cancer patients (all entities and stages) Adult patients (aged > 18) All sexes, all ethnicities	Patients with precancerous conditions or Carcinoma in situ Preclinical studies Primary Prevention Study populations with more than 20% children or precancerous conditions
Intervention	Every intervention with reflexology	
Comparison	All possible control groups (active, placebo, standard care, observation)	Other study types (one-armed/non-controlled studies, case reports or series)
Outcome	Mortality Morbidity Patient reported outcomes (with validated measurement tools) Symptoms measured with validated instruments Adverse effects	Laboratory parameters without diagnosis (except established surrogates for patient relevant outcomes; for example cortisol for stress)
Others	Meta-analyses, systemic reviews and RCTs Language: German and English Full publication	Grey literature (conference articles, abstracts, letters, ongoing studies, unpublished literature,…)

### Search and study selection

While searching for studies and selecting them, we followed the approach described in a systematic review by Römer et al. {Römer, 2021 #496}. A systematic research was conducted using five databases (Medline (Ovid), CINAHL (EBSCO), EMBASE (Ovid), Cochrane CENTRAL and PsycINFO (EBSCO)) in March 2022. For each of these databases a complex search strategy was developed consisting of a combination of MeshTerms, keywords and text words in different spellings connected to cancer and reflexology (Table 2). The search string was restricted by filters of study or publication type. After importing the search results into EndNote 20, all duplicates were removed and a title- abstract- screening was carried out by three independent reviewers (MK, JD, SK). In case of disagreement consensus was made by discussion or a fourth reviewer ^1^was consulted (JH). Furthermore, systematic reviews, which cover studies with a randomized controlled design were screened for relevant studies. When title and abstract did not have sufficient information for screening purposes, a full-text copy was retrieved as well. After that, all full texts were retrieved and screened again independently by both reviewers. Additionally, bibliography lists of all retrieved articles were searched for relevant studies.

**Table 2:** Search string reflexology - March 2022

Database	Search string
Ovid Medline	**1** exp Reflexotherapy/ or reflexolog$.mp. or reflexotherap$.mp **2** exp neoplasms/ or neoplasm$.mp or cancer$.mp. or tumo?r$.mp. or malignan$.mp. or oncolog$.mp. or carcinom$.mp. or leuk?emia.mp. or lymphom$.mp. or sarcom$.mp **3** 1 AND 2 **4** limit 3 to english or limit 3 to german **5** (4 and humans/) or (4 not animals/) **6** ((((comprehensive* or integrative or systematic*) adj3 (bibliographic* or review* or literature)) or (meta-analy* or metaanaly* or "research synthesis" or ((information or data) adj3 synthesis) or (data adj2 extract*))).ti,ab. or (cinahl or (cochrane adj3 trial*) or embase or medline or psyclit or (psycinfo not "psycinfo database") or pubmed or scopus or "sociological abstracts" or "web of science" or central).ab. or ("cochrane database of systematic reviews" or evidence report technology assessment or evidence report technology assessment summary).jn. or Evidence Report: Technology Assessment*.jn. or (network adj1 analy*).ti,ab.) or (((review adj5 (rationale or evidence)).ti,ab. and review.pt.) or meta-analysis as topic/ or Meta-Analysis.pt.) **7** Randomi?ed controlled trial?.pt. or controlled clinical trial?.pt. or randomi?ed.ti,ab.or placebo.ti,ab. or drug therapy.sh. or randomly.ti,ab. or trial?.ti,ab. or group?.ti,ab **8** 5 AND (6 OR 7) 9 5 NOT 8
Ovid Embase	**1** reflexology/ or reflexolog$.mp. or reflexotherap$.mp **2** exp neoplasm/ or neoplasm$.mp or cancer$.mp. or tumo?r$.mp. or malignan$.mp. or oncolog$.mp. or carcinom$.mp. or leuk?emia.mp. or lymphom$.mp. or sarcom$.mp **3** 1 AND 2 **4** limit 3 to english or limit 3 to german **5** (4 and humans/) or (4 not animals/) **6** ((((comprehensive* or integrative or systematic*) adj3 (bibliographic* or review* or literature)) or (meta-analy* or metaanaly* or "research synthesis" or ((information or data) adj3 synthesis) or (data adj2 extract*))).ti,ab. or (cinahl or (cochrane adj3 trial*) or embase or medline or psyclit or (psycinfo not "psycinfo database") or pubmed or scopus or "sociological abstracts" or "web of science" or central).ab. or ("cochrane database of systematic reviews" or evidence report technology assessment or evidence report technology assessment summary).jn. or Evidence Report: Technology Assessment*.jn. or (network adj1 analy*).ti,ab.) or (exp Meta Analysis/ or ((data extraction.ab. or selection criteria.ab.) and review.pt.)) **7** crossover procedure/ or double blind procedure/ or randomized controlled trial/ or single blind procedure/ or (random$ or factorial$ or crossover$ or (cross adj1 over$) or placebo$ or (doubl$ adj1 blind$) or (singl$ adj1 blind$) or assign$ or allocat$ or volunteer$).ti,ab,de **8** 5 AND (6 OR 7) **9** 5 NOT 8
Cochrane	**#1** [mh Reflexotherapy] or reflexolog* or reflexotherap*—709 **#2** [mh neoplasms] or neoplasm* or cancer? or tum*r? or malignan* or oncolog* or carcinom* or leuk*mia or lymphoma? or sarcoma?—271,683 **#3 #**1 AND #2—167
Ebsco—PsychINFO	**S1** reflexolog* or reflexotherap*—421 **S2** ((DE "Neoplasms" OR DE "Benign Neoplasms" OR DE "Breast Neoplasms" OR DE "Endocrine Neoplasms" OR DE "Leukemias" OR DE "Melanoma" OR DE "Metastasis" OR DE "Nervous System Neoplasms" OR DE "Terminal Cancer") OR (TX neoplasm* OR TX cancer OR TX tumo#r OR TX malignan* OR DE „oncology “ OR TX oncolog* OR TX carcinom* OR TX leuk#emia OR TX lymphoma OR TX sarcoma))—118,390 **S3** (LA German OR LA English)—4,920,144 **S4** S1 AND S2 AND S3—34 **S5** ((comprehensive* OR integrative OR systematic*) N3 (bibliographic* OR review* OR literature)) OR (meta-analy* or metaanaly* or "research synthesis" OR ((information OR data) N3 synthesis) OR (data N2 extract*)) OR ((review N5 (rationale OR evidence)) AND DE "Literature Review") OR (AB(cinahl OR (cochrane N3 trial*) OR embase OR medline OR psyclit OR pubmed OR scopus OR "sociological abstracts" OR "web of science" OR central)) OR DE "Meta Analysis" OR (network N1 analy*)—**283,546** **S6** DE "Treatment Effectiveness Evaluation" OR DE "Treatment Outcomes" OR DE "Psychotherapeutic Outcomes" OR DE "Placebo" or DE "Followup Studies" OR placebo* OR random* OR "comparative stud*" OR (clinical N3 trial*) OR (research N3 design) OR (evaluat* N3 stud*) OR (prospectiv* N3 stud*) OR ((singl* OR doubl* OR trebl* OR tripl*) N3 (blind* OR mask*)—550,013 **S7** S4 AND (S5 OR S6)—24 **S8** S4 NOT S7—10
Ebsco- CINAHL	**S1** MH Reflexology or reflexolog* or reflexotherap* **S2** (MH "Neoplasms + " OR TX neoplasm* OR TX cancer OR TX tumo#r OR TX malignan* OR TX oncolog* OR TX carcinom* OR TX leuk#emia OR TX lymphoma OR TX sarcoma) **S3** (LA German OR LA English) **S4** S1 AND S2 AND S3 **S5** (TI (systematic* n3 review*)) or (AB (systematic* n3 review*)) or (TI (systematic* n3 bibliographic*)) or (AB (systematic* n3 bibliographic*)) or (TI (systematic* n3 literature)) or (AB (systematic* n3 literature)) or (TI (comprehensive* n3 literature)) or (AB (comprehensive* n3 literature)) or (TI (comprehensive* n3 bibliographic*)) or (AB (comprehensive* n3 bibliographic*)) or (TI (integrative n3 review)) or (AB (integrative n3 review)) or (JN “Cochrane Database of Systematic Reviews”) or (TI (information n2 synthesis)) or (TI (data n2 synthesis)) or (AB (information n2 synthesis)) or (AB (data n2 synthesis)) or (TI (data n2 extract*)) or (AB (data n2 extract*)) or (TI (medline or pubmed or psyclit or cinahl or (psycinfo not “psycinfo database”) or “web of science” or scopus or embase)) or (AB (medline or pubmed or psyclit or cinahl or (psycinfo not “psycinfo database”) or “web of science” or scopus or embase or central)) or (MH “Systematic Review”) or (MH “Meta Analysis”) or (TI (meta-analy* or metaanaly*)) or (AB (meta-analy* or metaanaly*)) or network n1 analy* **S6** (MH "Clinical Trials + ") or PT Clinical trial or TX clinic* n1 trial* or TX ( (singl* n1 blind*) or (singl* n1 mask*)) or TX ((doubl* n1 blind*) or (doubl* n1 mask*)) or TX ( (tripl* n1 blind*) or (tripl* n1 mask*)) or TX ((trebl* n1 blind*) or (trebl* n1 mask*)) or TX randomi* control* trial* or (MH "Random Assignment") or TX random* allocat* or TX placebo* or MH "Placebos") or MH "Quantitative Studies") or TX allocat* random* **S7** S4 AND (S5 OR S6) **S8** S4 NOT S7

### Excluded studies

Excluded were 8 RCTs due to outcomes not being patient-relevant, patients not being cancer patients and multiple interventions. As the effects of the single parts of these interventions are not known and were not analyzed separately, it is not possible to estimate whether the reported effects are caused by the reflexology or by another treatment. A list of excluded studies can be seen in Appendix 1.

### Assessment of risk of bias and methodological quality

All characteristics were assessed by two independent reviewers (MK, JD). In case of disagreement a third reviewer was consulted (JH) and consensus was made by discussion.

The risk of bias in the included studies was analyzed with the Cochrane revised Risk of Bias Tool 2.0 [4].

Additional criteria concerning methodology were size of population, application of power analysis, adequacy of statistical tests (e.g. control of premises or multiple testing) and selective outcome reporting (report of all assessed outcomes with specification of statistical data as the *p*-value).

### Data extraction

Data extraction was performed by one reviewer (MK) and controlled by two independent reviewers (JD, JH). As a template for data extraction, the evidence tables from the National Guideline on Complementary and Alternative Medicine in Oncological Patients of the German Guideline Program in Oncology were used.

## Results

**Fig. 1:**
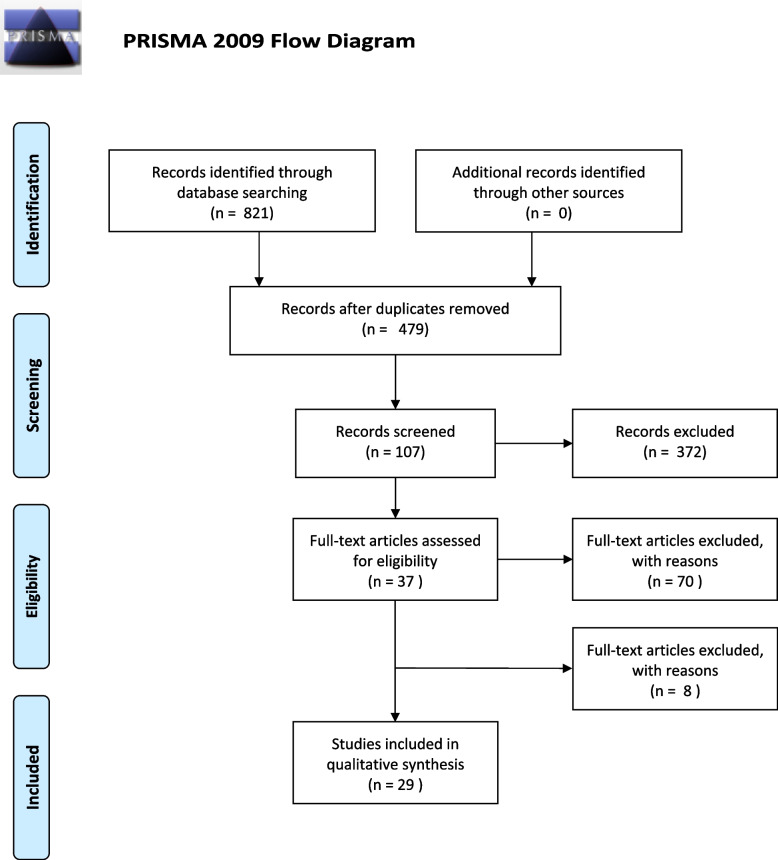
PRISMA flow diagram

The systematic search revealed 821 results. No studies were added by hand search. At first, duplicates were removed leaving 479 studies. After screening title and abstract, 133 studies remained to complete review (see Consort diagram, Fig. 1). Finally, 29 publications were analyzed in this review, including 29 RCT. According to this, the 29 publications reported data from 26 relevant studies. Detailed characterization of the included studies may be seen in Table 3.

**Table 3:** Short Evidence table

Reference	Study type	n/ cancer type/ Drop Out / m/f (%)	Intervention/ Duration	Endpoints	Outcomes
Mantoudi (2020)	RCT	*n* = 83 / Reflexology *n* = 40, Relaxation *n* = 40 / Dropout *n* = 3 / Cancer type: lung, prostate, urogenital, gastrointestinal / m (21%) vs. f (79%) Other therapies: [Arm A vs. B (%)]: Radiotherapy (32,5% vs. 52,5%), Chemotherapy (35% vs. 60%)	Reflexology vs. Relaxation 1 × 30 min per week for 6 weeks	1. Pain 2. Anxiety 3. Depression 4. Quality of life	1. Pain: Change from baseline to 4^th^ week: n.s Change from baseline to 6^th^ week: n.s 2. Anxiety: Change from baseline to 4^th^ week: n.s Change from baseline to 6^th^ week: n.s 3. Depression: Change from baseline to 4^th^ week: Reflexology -13.61 SD ± 10.93 and relaxation -6.49 SD ± 11.50, *p* = 0.006, Eta^2^ = 0.094 Change from baseline to 6^th^ week: Reflexology -19.58 SD ± 12.89 and relaxation -9.06 SD ± 13.76, *p* = 0.001, Eta^2^ = 0.138 4. Quality of Life: Physical component summary score: Change from baseline to 4^th^ week: n.s Change from baseline to 6^th^ week: Reflexology 20.75% SD ± 27.69 and relaxation 1.48% SD ± 13.23, *p* < 0.001, Eta^2^ = 0.168 Mental component summary score: Change from baseline to 4^th^ week: n.s Change from baseline to 6^th^ week: Reflexology 13.57% SD ± 14.93 and relaxation 5.72% SD ± 13.72, *p* = 0.017, Eta^2^ = 0.071
Göral Türkcü, Özkan (2021)	RCT	*n* = 68 / reflexology *n* = 31, control *n* = 31 / Dropout *n* = 6 / gynecological cancers / f (100%) Other therapies: Chemotherapy	Reflexology 3x/week for two weeks, 30–45 min Control group received standard care	1. Anxiety 2. Depression 3. Quality of life	1. Anxiety: Baseline: Reflexology 42.45 SD ± 9.49 vs. Control 43.77 SD ± 5.59, *p* = 0.71 After 4 weeks: Reflexology 32.94 SD ± 8.42 vs. Control 45.29 SD ± 5.32, *p* < 0.001 2. Depression: Baseline: Reflexology 36.26 SD ± 9,41 vs. Control 35.81 SD ± 7.70, *p* = 0.83 After 4 weeks: Reflexology 29.65 SD ± 8.51 vs. Control 40.52 SD ± 4.62, *p* < 0.001 3. Quality of Life: Global QoL: Baseline: Reflexology 46.77 SD ± Control 12.67 vs. 42.47 SD ± 11.05, *p* = 0.09 After four weeks: Reflexology 60.22 SD ± 17.17 vs. Control 40.59 SD ± 9.06, *p* < 0.01 Functional scale: Baseline: Reflexology 28.29 SD ± 12.68 vs. Control 23.10 SD ± 10.87, *p* = 0.08 After 4 weeks: Reflexology 39.53 SD ± 9.24 vs. Control 17.56 SD ± 6.99, *p* < 0.001 Symptoms scale: Baseline: Reflexology 71.02 SD ± 11.87 vs. Control 68.00 SD ± 12.21, *p* = 0.32 After four weeks: Reflexology 60.49 SD ± 7.01 vs. Control 72.66 SD ± 10.36, *p* < 0.001
Wyatt (2021)	RCT	*n* = 347 / Reflexology *n* = 150, Meditative Practices *n* = 150, Control *n* = 47 / Dropout *n* = 126 / Cancer type: breast, lung, colon, prostate, other / m (23%) vs. f (77%)	Caregivers were trained to provide reflexology (4x/week for 15 min) and meditative practices (4x/week for 30 min) at home After 4 weeks: nonresponding patients were randomized 1:1 to either the same group or the other group Responding patients continued their treatment for another four weeks Control group received standard care	1. Fatigue 2. Summed Symptom Severity 3. Anxiety and Depression	1. Fatigue: No significant group differences for all randomizations 2. Summed Symptom Severity: No significant group differences for all randomizations 3. Anxiety and Depression: No significant group difference for all randomizations
Murat-Ringot (2021)	RCT	*n* = 80 / reflexology *n* = 40, control *n* = 40, Dropout: *n* = 16 / cancer type: lung or digestive (stage II-IV) / sex (m/f) Reflexology: 67%/33% Control: 58%/42% Type of Chemotherapy: Carboplatin (moderately emetogenic) [Reflexology (37%), Control (37%)], Oxaliplatin (moderately emetogenic) [Reflexology (32%), Control (35%)], Cisplatin (highly emetogenic) [Reflexology (30%), Control (27%)]	Four sessions of reflexology (30 min each) every 2–3 weeks during chemotherapy infusion depending on the chemotherapy protocol for 4 cycles Control group received standard care	1.Chemotherapy induced nausea and vomiting (CINV) 2.Delayed CINV 3.Quality of Life 4.Anxiety 5.Self-esteem	1. CINV: per ITT: increase of VAS ≥ 2: reflexology 6/40 vs. control 13/40; *p* = 0.20 2. Delayed CINV: Across all cycles significant decrease in the use of antiemetic drugs (*p* = 0.04) Decrease in delayed nausea: n.s Difference in delayed vomiting: n.s 3. Quality of life: n.s 4. Anxiety: n.s 5. Average Body Image Score decrease: n.s
Sikorskii (2020)	RCT	*n* = 256, Reflexology *n* = 128, control *n* = 128, Dropout: *n* = 47, Breast cancer (stage III-IV), female Other therapies: Chemotherapy or targeted therapy (with or without hormonal): 188 patients Hormonal therapy only: 41 patients	Reflexology: receiving four weeks of home-based, lay caregiver–delivered (at least one per week) foot reflexology (following a 30-min foot reflexology protocol), and symptom assessment calls Control: receiving four weekly symptom assessment calls only	1.Severity of symptoms (pain, fatigue, disturbed sleep, shortness of breath, difficulty remembering, decreased appetite, dry mouth, numbness/tingling, vomiting/nausea) 2. Depression	1. Severity of symptoms: Significantly better results than attention control in pain [OR = 1.84, 95% CI (1.05, 3.23), *p* = 0.03] Any of the other symptoms: n.s 2. Depression: n.s The probability of intervention success was smaller with comorbidities [OR = 0.87, 95% CI (0.80, 0.94), *p* < .01] and higher CES-D at baseline [OR = 0.96, 95% CI (0.94, 0.99), *p* < .01] Older age was associated with significantly higher response rates to reflexology in terms of remembering [OR = 1.05, 95% CI (1.00, 1.11), *p* = 0.04] and disturbed sleep [OR = 1.06, 95% CI (1.01, 1.10), *p* = 0.02]
Sikorskii (2018)	RCT	*n* = 256, Reflexology *n* = 128, control *n* = 128, Dropout: *n* = 49, Breast cancer (stage III-IV), female	Reflexology: receiving four weeks of home-based, lay caregiver–delivered (at least one per week) foot reflexology (following a 30-min foot reflexology protocol), and symptom assessment calls Control: receiving four weekly symptom assessment calls only	1.Physical function 2.Pain 3.Fatigue 4.Sleep 5.Depression/mental health 6.Anxiety 7.Social functioning Comparison between different measurement tools	At Baseline values are given as Mean (SD) of all patients while at the follow up values are given as least square (SE) T0: Baseline T1: Week 5 (one week after intervention) 1.Physical function: n.s 2.Pain: T0: PROMIS-29 pain interference profile v1.0: (55.80 SD ± 9.47), SF-36 bodily pain (45.01 SD ± 10.35), PROMIS-29 pain severity profile v1.0 (3.60 SD ± 2.71), MDASI pain severity (3.57 SD ± 3.16) T1: PROMIS-29 pain interference profile v1.0: n.s SF-36 bodily pain: n.s PROMIS-29 pain severity profile v1.0: Significant difference between reflexology (2.87 SD ± 0.22) and control (3.51 SD ± 0.21), ES = 0.31, *p* = 0.04 MDASI pain severity: Significant difference between reflexology (2.09 SD ± 0.22) and control (3.06 SD ± 0.21), ES = 0.46, *p* < 0.01 3.Fatigue: T0: PROMIS-29 fatigue profile v1.0 (58.76 SD ± 8.98), SF-36 vitality (43.92 SD ± 10.29), MDASI fatigue severity (5.83 SD ± 2.73) T1: PROMIS-29 fatigue profile v1.0: n.s SF-36 vitality: n.s MDASI fatigue severity: Significant difference between reflexology (3.52 SD ± 0.24) and control (4.24 SD ± 0.23), ES = 0.31, *p* = 0.03 4.Sleep: n.s 5. Depression/mental health: T0: PROMIS-29 depression profile v1.0 (50.46 SD ± 8.10), CES-D (15.13 SD ± 10.52), MDASI sadness severity (3.22 SD ± 3.13), MDASI distress severity (3.80 SD ± 3.05), SF-36 mental health (49.62 SD ± 9.30) T1: PROMIS-29 depression profile v1.0: n.s CES-D: Significant difference between reflexology (11.76 SD ± 0.77) and control 814.09 SD ± 0.74), ES = 0.32, *p* = 0.03 MDASI sadness severity: n.s MDASI distress severity: Significant difference between reflexology (1.68 SD ± 0.23) and control (2.35 SD ± 0.22), ES = 0.31, *p* = 0.04 SF-36 mental health: Significant difference between reflexology (53.76 SD ± 0.76) and control (50.08 SD ± 0.73), ES = 0.51, *p* < 0.01 6.Anxiety: T0: PROMIS-29 anxiety profile v1.0 (52.69 SD ± 8.82), State anxiety (34.33 SD ± 11.67) T1: PROMIS-29 anxiety profile v1.0: Significant difference between reflexology (50.40 SD ± 0.71) and control (52.42 SD ± 0.67), ES = 0.30, *p* = 0.04 State anxiety: Significant difference between reflexology (30.93 SD ± 0.84) and control (34.05 SD ± 0.80), ES = 0.39, *p* = 0.01 7.Social functioning: T0: PROMIS-29 satisfaction with participation in social roles profile v1.0 (45.13 SD ± 8.40), SF-36 social functioning (43.79 SD ± 10.88) T1: PROMIS-29 satisfaction with participation in social roles profile v1.0: n.s SF-36 social functioning: Significant difference between reflexology (47.99 SD ± 0.88) and control (45.41 SD ± 0.85), ES = 0.31, *p* = 0.04
Samancioglu Baglama (2019	RCT	n = 64 / Dropout: *n* = 4 / Reflexology* n* = 32, Control (reading session) *n* = 32 / Cancer type: hematologic, breast, lung, genitourinary, gastrointestinal (stages I-IV) / Sex (m/f): 53,3% / 46,7% Other therapies: cyclophosphamide + doxorubicin/epirubicin + cyclophosphamide (38,3%), cyclophosphamide, methotrexate, 5-FU (33,3%), 5-FU + doxorubicin + cyclophosphamide/5-FU + epirubicin + cyclophosphamide; 5-FU, 5-fluorouracil (28,3%)	60-min teaching session for caregivers by a certified reflexologist Afterwards 60 min reflexology by caregivers a day and 60-min reading sessions for the control group for 15 days in total	1.pain 2.anxiety 3.fatigue	1.Pain No significant difference at the 1^st^ day between intervention (5.86 SD ± 2.16) and control (5.56 SD ± 0.95), *p* = 0.645 Significant difference at the 15^th^ day between intervention (4.70 SD ± 1.55) and control (6.36 SD ± 0.99), *p* = 0.000 The difference within the experimental group: n.s 2.Anxiety No significant difference at the 1^st^ day between intervention (6.20 SD ± 2.65) and control (6.26 SD ± 1.36), p = 0.891 Significant difference at the 15^th^ day between intervention (5.06 SD ± 1.59) and control (5.86 SD ± 1.27), *p* = 0.036 The difference within the experimental group was significant (*p* = 0.029) 3.Fatigue n.s The difference in the intervention group was significant (*p* = 0.005)
Rambod (2019)	RCT	n = 72 / Reflexology *n* = 36, Control *n *= 36 / Cancer type: Hodgkin- and Non-Hodgkin lymphoma / Sex (m/f): Reflexology (69,4%/30,6%), control (75%/25%) Other therapies (Intervention vs. control): Prior chemotherapy (77,8% vs. 69,4%), receiving chemotherapy (19,4% vs. 27,8%), post chemotherapy (2,8% vs. 2.8%)	Reflexology was provided by a certified reflexologist over 5 consecutive days (15 min on each foot) for the intervention group Control group received standard care	1.fatigue 2.pain 3.sleep	1. Fatigue Multidimensional fatigue inventory: No significant differences before between intervention (62.55 SD ± 11.27) and control (67.00 SD ± 12.70), *p* = 0.10 Significant differences after between intervention [53.41 (10.78)] and control [68.88 (12.48)], *p *< 0.001. Within group difference is also significant for intervention (*p* < 0,001) and nonsignificant for control (*p* = 0,08) General fatigue: No significant differences before between intervention (13.52 SD ± 3.37) and control (14.36 SD ± 3.39), *p *= 0.26, significant differences after: intervention [12.30 (3.21)] and control [14.33 (3.28)], *p* = 0.006 Physical fatigue: No significant differences before between intervention (13.63 SD ± 3.48) and control (14.08 SD ± 4.01), *p* = 0.57, but significant differences after: intervention [12.88 (3.69)] and control [14.91 (3.60)], *p* = 0.01 Mental fatigue: n.s Reduced activity: No significant differences before between intervention (14.88 SD ± 4.29) and control (15.80 SD ± 3.72), *p* = 0.29, but significant differences after: intervention [14.25 (3.36)] and control [16.25 (3,37)], *p* = 0.01 Reduced motivation: No significant differences before between intervention (7.05 SD ± 2.30) and control (8.00 SD ± 2.60), *p* = 0.11, but significant differences after: intervention [6.72 (2.22)] and control [7.83 (2.58)], *p* = 0.05 2. Pain No significant differences before between intervention (3.83 SD ± 2.79) and control (3.88 SD ± 3.46), *p* = 0.87, but significant differences after: intervention [2.72 (2.30)] and control [4.33 (3.54)], *p* = 0.01 3.Sleep Total sleep quality: Significant difference before between intervention (10.11 SD ± 3.26) and control (11.80 SD ± 3.83), *p* = 0.05 and after: intervention [8.41 (2.98)] and control [11.83 (3.26)], *p* < 0.001 Subjective sleep quality: No significant difference before between intervention (1.44 SD ± 0.74)] and control (1.63 SD ± 0.76), *p* = 0.25, but significant differences after: intervention [1.13 (0.42)] and control [1.69 (0.74)], *p* < 0.001 Sleep latency: No significant difference before between intervention (1.97 SD ± 0.99) and control (2.27 SD ± 0.74), *p* = 0.13, but significant differences after: intervention [1.58 (0.93)] and control [2.30 (0.78)], *p* = 0.001 Sleep duration: n.s Daytime dysfunction: n.s Sleep disturbance: No significant differences before between intervention (1.88 SD ± 0.57) and control (1.86 SD ± 0.72), *p* = 0.84, but significant differences after: intervention [1.44 (0.50)] and control [1.80 (0.66)], *p* = 0.01 Sleep medication: n.s Sleep sufficiency: Significant differences before between intervention (0.72 SD ± 1.11) and control (1.27 SD ± 1.20), *p* = 0.03 and after: intervention [0.52 (.84)] and control [1.36 (1.22)], *p* < 0.001
Nourmohammadi (2019)	RCT	*n* = 60 / Reflexology *n* = 30, Control *n* = 30 / Dropout: *n* = 3 / Cancer type: breast cancer (stage I) / female	Reflexology group received intervention 2x/week (20 min per session) for four consecutive weeks Control group received standard care	1.fatigue	1. Fatigue No significant differences in fatigue before the intervention between reflexology (45.44 SD ± 5.30) and control (43.66 SD ± 7.68), *p* = 0.31 Follow up two months after the intervention: significant difference between reflexology (20.66 SD ± 4.54) and control (40.36 SD ± 9.58), *p* = 0.000 Difference within the reflexology group: significant (*p* = 0.000) Difference within the control group: nonsignificant (*p* = 0.16)
Hesami (2019)	RCT	*n* = 80 / Reflexology *n* = 40, Control *n* = 40 / Cancer type: Digestion system, blood & lymph system, other / Sex (m/f) Intervention ( 50%/50%), Control (45%/55%)	Foot reflexology for four consecutive days (30 min per session) Control group received standard care	1.fatigue	1. Fatigue: Before intervention: No significant difference between intervention (5.538 SD ± 1.041) and control (5.000 SD ± 1.398), *p* = 0.054 After the intervention: Significant difference in the intervention group (4.486 SD ± 1.040, *p* = 0.000) Significant difference in fatigue in the control group (5.180 SD ± 1.450, *p* = 0.036) Significant difference between groups after the intervention (*p* = 0.016)
Dikmen (2019)	RCT	*n* = 140 / Intervention groups *n* = 100, Control *n* = 40 / Dropout: *n *= 60 / Cancer types: Uterine, ovarian, cervical (grade I-III) / no information on sex Other therapies: 2^nd^ or 3^rd^ cycle of chemotherapy	Reflexology: 2x/week (30 min per session) for 8 weeks applied by the researcher Progressive Muscle Relaxation Exercises: 2x/week (20 min per session) for 8 weeks under supervision of the researcher Reflexology + Progressive Muscle Relaxation Control group received standard care	1.pain 2.fatigue 3.quality of life	T1: Admission to hospital (baseline) T2: week 3 T3: week 8 T4: week 12 (follow up) 1.Pain Significant differences between groups at baseline for pain severity (*p* = 0.001) and insignificant differences for the effects of pain on daily life (*p* = 0.225) Significant differences at 3^rd^ week for pain severity (*p* = 0.001) and the effects of pain on daily life (*p* = 0.001) Significant differences at 8^th^ week for pain severity (*p* = 0.001) and the effects of pain on daily life (*p* = 0.001) Significant differences at 12^th^ week for pain severity (*p* = 0.013) and the effects of pain on daily life (*p* = 0.017) 2.Fatigue Insignificant differences between groups at baseline for fatigue severity (*p* = 0,218) and effects of fatigue on daily life (*p* = 0,065) Significant differences at 3^rd^ week for fatigue severity (*p* = 0,001) and effects of fatigue on daily life (*p* = 0,001) Significant differences at 8^th^ week for fatigue severity (*p* = 0,001) and effects of fatigue on daily life (*p* = 0,001) Significant differences at 12^th^ week for fatigue severity (*p* = 0.039) and effects of fatigue on daily life (*p* = 0.001) 3.Quality of Life Insignificant differences between groups at baseline (*p* = 0.079) Significant differences at 3^rd^, 8^th^ and 12^th^ week (*p* < 0.05)
Rezaei (2021)	RCT	*n* = 66 / Reflexology *n* = 33, Control *n* = 33 / Cancer Type: Breast cancer (stage 1–4) / Sex: female Current therapies: Chemotherapy (85.35%), chemotherapy and radiotherapy (48.48%)	Reflexology applied for 40 min twice in one day (morning and afternoon) Control group received standard care	1.anxiety	1. Anxiety n.s
Jahani (2018)	RCT	*n* = 84 / Reflexology *n* = 42, Control *n* = 42 / Cancer type: Hematologic cancer with metastases / Sex (m/f): Reflexology (54,8% / 45,2%), Control (54,8% / 45,2%)	Reflexology performed on one day Control group received standard care	1.pain 2.anxiety	1. Pain T1: 3 days before treatment T2: 2 days before T3: 1 day before T4: 1 day after treatment T5: 2 days after T6: 3 days after T1: No significant difference between reflexology (5.86 SD ± 2.46) and control (5.48 SD ± 2.50), *p* = 0.45 T2: p-value not given T3: Significant difference between reflexology (4.12 SD ± 2.18) and control (6.57 SD ± 2.08), *p* = 0.001 T4: p-value not given T5: Significant difference between reflexology (3.88 SD ± 2.039) and control (5.67 SD ± 1.946), *p* = 0.001 T6: Significant difference between reflexology (2.83 SD ± 1.793) and control (6.4 SD ± 1.835), *p* = 0.001 Intervention group: significant difference between first day of testing and last day of testing (*p* = 0.001) 2.Anxiety T1:1 day before treatment T2: 3^rd^ day after treatment T1: No significant differences between reflexology group (46.62 SD ± 18.314) and control group (44.69 SD ± 18.296), *p* = 0.59 T2: Significant difference after the treatment between reflexology (41.76 SD ± 17.442) and control group (44.29 SD ± 18.311), *p* = 0.04 Intervention group: Significant improvement to before the intervention (*p *= 0.008) Control group: n.s
Kurt (2018)	RCT	*n* = 96 / Reflexology *n* = 46, Control *n *= 50 / Dropout: *n* = 36 / Cancer type: Breast, digestive system, other cancer / Sex (m/f): Reflexology (53,3% / 46,7%), Control (53,3% / 46,7%) Chemotherapy treatment: Eloxatin-based, Taxan-based, Platin-based, Taxan-Platin-based, Fluoracil-based	Reflexology 2x/day (20 min per session) for six weeks Control group received standard care	1.quality of life 2.pain	T1: at first meeting T2: after 3 weeks T3: after 6 weeks 1.Quality of life: Sensory function: T1: No significant difference between Intervention (37.77 SD ± 19.46) and control (40.49 SD ± 21.87), *p* = 0.78 T2: n.s T3: Significant difference between intervention (22.83 SD ± 16.50) and control (34.44 SD ± 20.77), *p* = 0.024 Motor function: n.s Autonomic function: n.s 2.Pain General Activity: n.s Walking ability: n.s Normal Work: n.s Relations with other people: n.s Sleep: n.s Enjoyment of life: n.s
Sharp (2010)	RCT	*n* = 183 / Reflexology *n* = 60, Scalp Massage *n* = 61, SIS *n* = 62 / Dropout *n* = 17 / Cancer type: Breast cancer: T1, T2 (< 3cm), N0, N1a, M0 / Sex: 100% female Current therapies: RTX *n* = 193, CTX *n* = 30	Arm A: Reflexology 1h 1x/week for 8 weeks + SIS (self-initiated support) Arm B: Scalp Massage 1h 1x/week for 8 weeks + SIS Arm C: SIS	1.Quality of Life (at T1) 2.Quality of Life (at T2) 3.Relaxation Scale with Mood Rating Scale 4. Other Scales with MRS 5.Quality of Life (physical, functional, emotional, social, additional concerns scales) 6.Anxiety and Depression 7.Complementary Therapies Questionnaire 8.Psychiatric Morbidity	T1: week 18 post surgery T2: week 24 post surgery [Mean (95% CI)] 1.Quality of Life at T1: Arm B significantly better than Arm C [Arm B: 73.06 (70.89,75.23), Arm C: 69.05 (66.90,71.21); *p* = 0.03] Other arms: n.s 2. Quality of Life at T2: Arm A significantly better than Arm C [Arm A: 74.82 (72.13,77.55), Arm C: 69.42 (66.75,72.09); *p* = 0.02] Other arms: n.s 3.Relaxation Scale with MRS: T1: Significantly better results for Arm A and B vs. Arm C [Arm A: 100.94 (91.36,110–53), Arm B: 100.23 (90.77,109.69), Arm C: 69.05 (66.90,71.21); p(AC) < 0.0005, p(BC) < 0.0005] T2: Significantly better results for Arm A vs. Arm C [Arm A: 107.30 (97.91,116.69), Arm C:89.07 (79.82,98.32); *p* = 0.02] Other arms: n.s 4.Other scales with MRS: T1: Significantly better results on “easy-goingness” scale for Arm A vs. Arm B and C [Arm A: 98.70 (90.12,107.27), Arm B: 113.98 (105.49,122.46), Arm C: 89.18 (80.73,97.63), p(AB) = 0.04, *p*(BC) < 0.0005] 5.Quality of Life: T1: n.s T2: Significantly better results for Arm A vs. C in total score and “functional wellbeing” subscale [total score: Arm A: 118.60 (114.93,112.26), Arm C: 111.70 (108.10,115.30); *p* = 0.03, „functional wellbeing “: Arm A: 23.17 (22.01,24.33), Arm C: 21.04 (19.90,22.17); *p* = 0.03] Other arms: n.s 6. Anxiety and Depression: n.s 7.Complementary Therapies Questionnaire: n.s 8. Psychiatric Morbidity: n.s
Stephenson (2007)	RCT	*n* = 90 / Analysed: Reflexology *n* = 42, Control *n* = 44 / Dropout n = 4 / Cancer type: lung, breast, colorectal, head and neck, lymphoma / Sex: 51% female Other therapies: CTX, RTX, surgery (at least 6 weeks ago)	Arm A (Reflexology): 1 × 30 min by partner/family member (taught by certified reflexologist) Arm B (Control): 1 × 30 min reading session by partner/family member	1.pain (BPI) 2.pain (McGill Pain Questionnaire) 3.anxiety	(adjusted mean difference pre/post) 1. or 2. (not clear) Pain: Significantly better results for Arm A vs. B (Arm A: 1.1, Arm B: 0.1; *p* = 0.001; = 0.12) Subgroup analysis: Significantly better results for Arm A vs. B for patients wit pain > 5 (Arm A: 2.7, Arm B: 0.5; *p* = 0.007; = 0.23) 3.Significantly better results for Arm A vs. B (Arm A: 3.1, Arm B: 1.3; *p* = 0.001; = 0.13) Subgroup analysis: Significantly better results for Arm A vs. B for all patients with anxiety > 5 (Arm A: 5.0, Arm B: 2.5; *p *= 0.006; = 0.15)
Dyer (2013)	RCT	*n* = 115 / Aromatherapy massage *n* = 58, Reflexology *n* = 57 / Dropout *n* = 11 / Cancer type: breast, gastrointestinal, gynaecological, haemato-oncology, head and neck, lung, neurology, sarcoma, skin, urology, cancer of unknown primary / Sex: 93% female Other therapies: Aromatherapy massage: 17 CTX, 41 other, Reflexology: 14 CTX, 43 other	Arm A (aromatherapy massage): 4 × 1h (median length between first and last treatment: 10 weeks) Arm B (reflexology): 4 appointments (no further information)	1.MYCaW (first concern scores) 2.MYCaW (second concern scores) 3.MYCaW (overall wellbeing scores) 4.change over time (from first to fourth session) in pre-session VAS relaxation score 5.change in pre to post-session VAS relaxation score 6.percentage of patients gaining benefit from the intervention 7.MYCaW follow up form patient’s written comments	1.MYCaW (first concerns): Mean difference 0.453 (SE = 0.323) in favor of aromatherapy (*p* = 0.046) 2.MYCaW (second concerns): n.s 3.MYCaW (overall wellbeing score): n.s 4.Change over time in pre session VAS relaxation score: n.s 5.Change in pre to post session VAS relaxation score: n.s 6.Patients gaining benefit from the intervention: n.s 7.Most frequent answer for „What has been most important for you?” was “Relaxation and time for oneself” in both arms
Hodgson (2012)	RCT	*n *= 18 / Arm A = NI, Arm B = NI / Dropouts *n* = 0 / Cancer type: breast, prostate, colorectal, lung / Sex: 66% female Other therapies: Cancer treatment completed	Arm A: “friendly visits” for baseline assessments (week 1), Swedish massage of lower extremities (20 min 1x/week for 4 weeks); 1 week washout; foot reflexology (20 min 1x/week for 4 weeks) Arm B: “friendly visits” for baseline assessments (week 1), foot reflexology (20 min 1x/week for 4 weeks); 1 week washout; foot Swedish massage of lower extremities (20 min 1x/week for 4 weeks)	1.5-min observation of affect (positive mood) 2. 5-min observation of affect (negative mood) 3.pain	1.5-min observation of affect (positive mood): n.s 2. 5-min observation of affect (negative mood): n.s 3.Pain: n.s
Özdelikara (2017): The Effect of Reflexology on Chemotherapy-induced Nausea, Vomiting, and Fatigue in Breast Cancer Patients	RCT	*n* = 60 / Arm A: Reflexology *n* = 30, Arm B: Control / Dropout: NI / Cancer type: breast (stage I-III) / Sex: 100% female Other therapies: CTX (Epirubicin/ Cyclophosphamid)	Arm A: Reflexology during 2^nd^ to 4^th^ CTX cycle (30–40 min during CTX) Arm B: CTX plus usual care	1a. INVR: Subscale of experience 1b. INVR: Subscale of symptom development 1c. INVR: Subscale of distress development 2a. Fatigue severity 2b. Daily life activity exposure levels	Baseline: within 24h after first CTX cycle Posttreatment Assessments: 24h after 2^nd^ to 4^th^ CTX cycle Results of last assessment (24h after 4^th^ CTX cycle) shown: 1a: INVR: subscale of experience: n.s 1b: INVR: subscale of symptom development: Significant difference of both arms to baseline (Mean [SD]: Arm A: 11.10 [4.74], Arm B: 6.76 [6.85]; *p* = < 0.05) Significant difference at last assessment between arms (Mean [SD]: Arm A: 2.50 [4.34], Arm B: 9.00 [5.29]; *p* = 0.000) 1c: INVR: subscale of distress development: Significant difference of both arms to baseline (Mean [SD]: Arm A: 6.90 [2.90];, Arm B: 4.2 [4.47]: *p* = < 0.05) Significant difference at last assessment between arms (Mean [SD]: Arm A: 1.40 [2.59];, Arm B: 5.73 [3.55]; *p* = 0.000) 2a: Fatigue severity: Significant difference of both arms to baseline (Mean [SD]: Arm A: 3.67 [1.94], Arm B: 1.97 [1.59]; *p* = 0.000) Significant difference at last assessment between arms (Mean [SD]: Arm A: 1.20 [1.44], Arm B: 2.33 [1.65]; p < 0.05) 2b: Daily life activity exposure levels: Significant difference of both arms to baseline (Mean [SD]: Arm A: 1.88 [1.26], Arm B: 1.01 [1.16]; *p* = < 0.05) Significant difference at last assessment between arms (Mean [SD]: Arm A: 0.41 [0.65], Arm B: 1.47 [1.52]; *p* = 0.001)
Özdelikara (2017): The effect of reflexology on the quality of life with breast cancer patients	RCT	*n* = 60 / Arm A: Reflexology *n* = 30, Arm B: Control / Dropout: NI / Cancer type: breast (stage I-III) / Sex: 100% female Other therapies: CTX (Epirubicin/ Cyclophosphamid)	Arm A: Reflexology during 2^nd^ to 4^th^ CTX cycle (30–40 min during CTX) Arm B: CTX plus usual care	1a. EORTC-QLQ-C30: General Health Score 1b: EORTC-QLQ-C30: Function Score 1c: EORTC-QLQ-C30: Symptom Scale	Baseline: within 24h after first CTX cycle Posttreatment Assessments: 24h after 2^nd^ to 4^th^ CTX cycle 1a: EORTC-QLQ-C30: General Health Score: Pretest: Mean (SD): Arm A: 55.55 (24.79), Arm B: 54.16 (21.74); *p* = NI Posttest: Mean (SD): Arm A: 78.61 (13.43), Arm B: 31.66 (18.62); *p* = 0.000 1b: EORTC-QLQ-C30: Function Score: Pretest: Mean (SD): Arm A: 71.25 (15.17), Arm B: 80.29 (14.32); *p* = NI Posttest: Mean (SD): Arm A: 89.92 (6.51), Arm B: 64.07 (17.52); *p *= 0.000 1c: EORTC-QLQ-C30: Symptom Scale: Pretest: Mean (SD): Arm A: 35.81 (14.35), Arm B: 21.02 (18.53); *p* = NI Posttest: Mean (SD): Arm A: 9.31 (7.54), Arm B: 39.23 (15.22); *p* = 0.000
Stephenson (2000)	RCT	*n* = 24 / Dropout *n* = 1 / Cancer type: breast, lung / Sex: 65% female Other therapies: NI	Arm A: Reflexology (1 × 30 min), at least 48h in between, passive control/no intervention (1 × 30 min) Arm B: passive control/no intervention (1 × 30 min), at least 48h in between, reflexology (1 × 30 min)	1.anxiety 2.pain with SF-MPQ 3.pain intensity with SF-MPQ: PPI-scale 4.pain intensity with VAS	1.Anxiety: Significantly better results after intervention compared to control (Mean difference = -21.83; *p* < 0.000) 2. Pain with SF-MPQ: Only breast cancer patients analysed: Significantly better results after intervention compared to control (Mean difference = -0.41; *p* < 0.05) 3. Pain intensity with SF-MPQ: PPI-scale: Only breast cancer patients analysed: n.s 4. Pain intensity with VAS: Only breast cancer patients analysed: n.s
Tsay (2008)	RCT	n = 62 / Reflexology *n* = 30, Control *n* = 31 / Dropout *n* = 1 / Cancer type: hepatocellular, gastric / Sex: 52.46% female	Arm A: Surgery + Reflexology (3 days following surgery) for 20 min Arm B: Surgery	1.pain with VAS (baseline, T1-T3, follow up) 2.pain with SF-MPQ (baseline, follow up) 3.anxiety (baseline, follow up) 4.narcotic analgesia consumption (Demerol)	Baseline T0: day 2 surgery, T1-T3: day 2–4 surgery, Follow-up: day 5, 6 surgery 1.Pain with VAS: All points in time: On average significantly better (lower) results in Arm A compared to Arm B (β_G_ = -21.22 (SE = 4.93); *p* < 0.001) 2.Pain with SF-MPQ: All points in time: On average no significant differences 3.Anxiety: All time points: On average no significant differences 4.Narcotic analgesia consumption: Significantly lower consumption in Arm A (mean = 39.59mg Demerol; *p* = 0.015)
Uysal (2017)	RCT	*n* = 65 / Reflexology *n* = 21, Classical Massage *n* = 22, Control *n* = 22 / Dropout *n* = 5 / Cancer type: Colorectal (stage II and III) / Sex: 50% female Other therapies: CRT (5 weeks): CTX 5-Fluoruracil/ Capecitabine + RTX (1.8‐2 Gy/5 days)	Arm A: Reflexology (30 min 2x/week for 5 weeks in total) + CRT (chemoradiotherapy) Arm B: Classical Massage (20 min 2x/week for 5 weeks in total) + CRT Arm C: CRT + usual care	1.QoL with EORTC QLQ C30 (function scale, symptom scale, global health scale) 2.QoL with EORTC QLQ CR29 3.Adverse effects	Measurements were taken at week 1, 3 and 5 1. QoL with EORTC QLQ C30: Function scale: Significantly better results in Arm A compared to Arm C in week 3 (Mean [SD]: A: 82.66 [4.42], C: 80.22 [8.64]; *p* < 0.000) and in week 5 (A: 81.98 [4.79]; C: 71.66 [9.34]; *p* < 0.000) Symptom scale: Significantly better results in Arm C compared to A in week 3 (Mean [SD]: A: 21.66 [22.36]; C: 20.00 [16.75]; *p* = 0.003) Significantly better results in Arm A compared to C in week 5 (A: 25.00 [32.21], C: 31.66 [29.56]; *p* < 0.000) Significantly better results in Arm B compared to C in week 5 (B: 26.66 [13.67]; C: 31.66 [29.56]; *p* < 0.000) Global health scale: Significantly better results in Arm A compared to C or B in week 1 (Mean [SD]: B: 68.33 [5.72], A:76.25 [9.85],,C: 68.33 [11.34]; *p* = 0.012) Significantly better results in Arm A compared to C or B in week 3 (B: 61.57 [8.56], A: 70.55 [8.56], C: 60.67 [8.76]; *p* < 0.000) Significantly better results in Arm A compared to C or B in week 5 (B: 57.08 [9.07], A: 69.16 [9.40], C: 54.16 [9.55]; *p* < 0.000) 2. QoL with EORTC QLQ CR29: No information 3.Adverse effects: Significantly less pain with grade 2 + in Arm A and B compared to Arm C in week 4 (A: 19.3%, B: 30.7%, C 50%; *p* = 0.002) Significantly less pain with grade 2 + in Arm A and B compared to Arm C in week 5 (A: 16,2%, B:35,4%, C: 43,4%; *p* < 0.001) Significantly less fatigue with grade 2 + in Arm A compared to Arm B and C in week 3 (A: 28%, B:36%, C:36%; *p* = 0.03) Significantly less fatigue with grade 2 + in Arm A compared to Arm B and C in week 4 (A: 28,6%, B:35,7%, C: 35,7%; *p* < 0.001) Significantly less fatigue with grade 2 + in Arm A compared to Arm B and C in week 5 (A: 30,4%, B:34,8%, C:34,8%; *p* = 0.036) Significantly lower urinary frequency with grade 1 + in Arm A compared to Arm C in week 5 (A:25%, C:37,5%; *p* = 0.044) Significantly less distension with grade 1 + in Arm A compared to C in week 4 (A:15,2%, C:42.4%; *p* < 0.000) and 5 (A:20%, C:56%; *p* < 0.000) Significantly more distension with grade 1 + in Arm B compared to C in week 2 (B:44.1%,C: 20.6%; *p* = 0.033) Significantly less distension in with grade 1 + in Arm B compared to C in week 5 (B: 24%, C:56%; *p* < 0.000) No significant differences between groups for nausea, vomiting, constipation, diarrhea and proctitis
Wyatt (2012)	RCT	*n* = 286 / Reflexology *n* = 95, Foot Massage *n* = 95, Control *n *= 96 / Dropout *n* = 27 / Cancer type: breast / Sex: 100% female Other therapies: Chemotherapie and/or hormonal therapy	Arm A: Reflexology 1x/week (30 min) for 4 weeks Arm B: Foot massage 1x/week (30 min) for 4 weeks Arm C: Control with conventional therapy	1.Breast Cancer specific QoL with subscales (physical, emotional, social, functional) 2.Dyspnea 3.nausea 4.physical function 5.fatigue 6.interference of fatigue with activities of daily living 7.pain intensity 8.depression 9.anxiety	T0: Baseline T1: 1 week after intervention T2: 6 weeks after intervention 1.Breast Cancer specific QoL: n.s 2.Dyspnea: Significantly better outcome in Arm A compared to C (β = 0.39 [0.13]; *p* < 0.01, Arm A: M-T1 = 3.33, M-T2 = 3.36, Arm C: M-T1 = 3.1 M-T2 = 2.9, Effect T1 = 0.36, Effect T2 = 0.51) Significantly better outcome in Arm A compared to B (β = NI); *p* = 0.02, Arm B: M-T1 = 3.1, M-T2 = 3.03, Effect T1 = k.A., Effect T2 = NI) 3.Nausea: n.s 4.Physical function: Significantly better (higher) results for Arm a compared to C (ß = 5.527 [2.728]; *p* = 0.04, Arm A: M-T1 = 58.6, M-T2 = 59.2, Arm C: M-T1 = 54.9 M-T2 = 51.6, Effect T1 = 0.21, Effect T2 = 0.44) 5.Fatigue: n.s 6. Interference of fatigue with daily activities: n.s 7. Pain intensity: n**.**s 8. Depression: n.s 9. Anxiety: n.s
Wyatt (2017)	RCT	*n* = 256 / Reflexology *n* = 128, Attention Control *n* = 128 / Dropout *n* = 76 / Cancer type: breast cancer (stage III-IV) / Sex: 100% female Other therapies: Chemotherapy or targeted therapy, hormonal therapy	Arm A: Reflexology by caregiver (30 min/1x/week for 4 weeks) Arm B. Attention Control Calls (1x/week for 4 weeks)	1.Summed symptom severity 2.Interference of symptoms with daily life 3.Physical functioning 4.Satisfaction with participation in social roles 5.Quality of Life 6.Perceived social support 7.Quality of relationship between patient and caregiver	1.Summed symptom severity: Significantly better (lower) results for Arm A compared to B (β = -4.34 [SE = 1.85]; p = 0.02) in week 2,3 and 5 (week 2: Arm A: M = 27.50 [SD = 1.53], Arm B M = 33.65 [SD = 1.55]; *p* < 0.01; week 3: Arm A: M = 25.50 [SD = 1.55], Arm B: M = 30.98 [SD = 1.55]; *p* = 0.01; week 5: Arm A: M = 24.64 [SD = 1.52], Arm B: M = 30.50 [SD = 1.48]; *p* < 0.01) 2. Interference of symptoms with daily life: Significantly better (lower) results for Arm A compared to B (β = -3.69 [SE = 1.39]; *p* < 0.01) in week 2,3 and 5 (week 2: Arm A: M = 14.60 [SD = 1.15], Arm B: M = 18.32 [SD = 1.17]; *p* = 0.02; week 3: Arm A: M = 11.84 [SD = 1.17], Arm B: M = 17.57 [SD = 1.17]; *p* < 0.01; week 5: Arm A: M = 12.30 [SD = 1.15]; Arm B: M = 16.60 [SD = 1.12]; *p* < 0.01) 3. Physical functioning: n.s 4. Satisfaction with participation in social roles: n.s 5.Quality of life: n.s 6. Perceived social support: n.s 7. Quality of relationship between patient and caregiver: n.s
Anderson (2021)	RCT	*n* = 40 / Reflexology *n* = 20, Control *n* = 20 / Dropout *n* = 0 / Cancer type: Leukemia, lymphoma, brain, colon, multiple myeloma, lung, ovarian, sarcoma, pancreatic, other / Sex: 63% female Other therapies: Chemotherapy, Radiotherapy, other	Arm A: 1 Reflexology session of 20–25 min Arm B: 1 session of 20–25 min during which surveys were administered	1.pain 2.nausea	1.Significant improvement for intervention group from pre to post session (*p* < 0.0001) but not for control 2.No significant improvement for nausea in the intervention group from pre to postsession (*p* = 0.06)
Ross (2002)	RCT	*n* = 26 / Reflexology *n* = 12, Foot Massage *n* = 14 / Dropout *n* = 9 / Cancer type: advanced cancer / Sex (only evaluated patients): 4 males, 13 females Other therapies: No ongoing anticancer therapies	Arm A: Reflexology 1x/week for 6 weeks Arm B: Basic foot massage 1x/week for 6 weeks	1.Anxiety and Depression 2.Symptom distress score	1.Anxiety and depression: No difference in the reflexology group from baseline [14.57 (SD 2.87)] to week 6 [14.29 (SD 2.59)] No difference in the massage group from baseline [13.90 (SD 1.82)] to week 6 [13.20 (SD 2.97)] 2.Symptom distress score: No difference between groups except a significantly greater improvement in appetite and mobility in the foot massage group
Stephenson (2003)	RCT	*n* = 36 / Reflexology *n* = NI, Control *n *= NI / Dropout *n* = NI / Cancer type: lung, lymphoma, colorectal, other (all metastatic) / Sex: 55.6% female Other therapies: NI except opioid analgesics (parenteral morphine equivalent)	Arm A: Reflexology 2 × 24 h apart Arm B: Control	1.Pain	1.Pain: Directly after intervention: significantly lower pain levels in the intervention group compared to control (F[1, 29] 9.08, *p* < 0.01) No significant effect at 3 h after (*p* = 0.21) and at 24 h after (*p* = 0.14) intervention
Hodgson (2000)	RCT	*n* = 12 / Reflexology *n* = 6, Placebo Reflexology *n* = 6 / Dropout n = 0 / Cancer type: various / Sex: 58% male Other therapies: NI	Both arms: roughly 40 min of either reflexology or placebo reflexology on day 1, 3 and 5 of hospital stay	1.Quality of life	1. significant differences in favor of the reflexology group post test for breathing with a mean improvement of 2.2 points on VAS (*p* = 0.026) and overall (*p* = 0.004)

*Abbreviations: SD:* Standard Deviation, *CI* Confidence Intervall, *SE* Standard Error, *SEB* Standard Error of β, *OR* Odds Ratio, *NI* No information, *MP* Meditative Practices, *ITT* Intention-to-treat-anaylsis, *CES-D* Center for Epidemiologic Studies Depression Scale, *CINV* Chemotherapy-induced nausea, *CTX* Chemotherapy, *EORTC QLQ* European Organization for the Research and Treatment of Cancer Quality of Life Questionnaire, *MDASI* M.D. Anderson symptom inventory, *MRS* Mood rating scale, *MYCaW* Measure Yourself Concerns and Wellbeing, *PROMIS* Patient Reported Outcomes Measurement System, *QoL* Quality of Life, *SF-MPQ* Short Form-McGill Pain Questionnaire, *SIS* Self-initiated support, *VAS* Visual analogue scale

### Characteristics of included studies

Concerning all relevant studies, 2465 patients were included and 2262 of them were analyzed, due to 405 drop outs. The age of patients ranged from 18 to 98 years. 70.8% of the participants were female. Endpoints these studies investigated include pain, anxiety and depression, fatigue, QoL/symptom severity and distress, physical and social functioning/interference with daily life, nausea and vomiting, sleep, mood, relaxation, narcotic analgesia consumption, self-esteem, psychiatric morbidity, perceived social support and quality of relationship between caregiver and patient. While physical and social functioning/interference with daily life could also be counted towards QoL, for the sake of clarity we decided to report them separately.

### Risk of bias in included studies

The methodical quality was assessed with the Cochrane revised Risk of Bias Tool 2.0 [4]. The results are presented in Table 4. Three of the included studies show moderate risk of bias and 26 show high risk of bias.

**Table 4:** Risk of Bias Assessment

Reference	Study type	Standardized rating of risk of bias	Additional comments on methodology
Mantoudi (2020)	RCT	RoB Randomised Assignment: low Deviations from the intended interventions: some concern Missing outcome data: low Measurement of the outcome: high Selection of the reported result: low Overall Risk of Bias: high	PRO: Methodical quality: randomization by independent person, testing for normal distribution, controlling for multiple testing, active control group Report quality: specification of effect sizes, information on other therapies CONTRA: Methodical quality: No homogeneity for chemotherapy between groups at 5% significance level (In order to reach homogeneity, significance level was decreased to 1% for this parameter), no power analysis, researcher conducted interventions, no blinding Report quality: No information where researcher learned how to apply reflexexology, No comparison of drug dosage at baseline, no specification on comorbidities
Göral Türkcü, Özkan (2021)	RCT	RoB Randomised Assignment: some concern Deviations from the intended interventions: high Missing outcome data: low Measurement of the outcome: high Selection of the reported result: low Overall Risk of Bias: high	PRO: Sample: Homogeneity between arms Methodical quality: Approved by ethics committee, randomization via SPSS, power analysis, testing for normal distribution, Mann–Whitney U test for data without normal distribution, control for multiple testing CONTRA: Sample: only gynecological cancers (possibly limited carryover to other patient groups) Methodical quality: single blind (not possible), side effects only reported by researchers based on verbal and non-verbal responses of the patients, researcher applied intervention and collected data, no information on comorbidities, therapies or medication, short time frame of intervention Report quality: no information if researcher had formal training, no data on control group after 2^nd^ week
Murat-Ringot (2021)	RCT	RoB Randomised Assignment: some concern Deviations from the intended interventions: low Missing outcome data: low Measurement of the outcome: high Selection of the reported result: high Overall Risk of Bias: high	PRO: Sample: Homogeneity between groups Methodical quality: Intention-to-treat analysis for primary endpoint, power analysis, Sensitivity analyses for patients without VAS assessments during the 2^nd^ cycle of chemotherapy, Categorical variables compared between groups, Comparison of nonparametric variables, reflexology applied by three certified reflexologists CONTRA: Methodical quality: no blinding, initially planned statistical method was altered, per protocol analysis for secondary outcome, for self-esteem two different scales were used at baseline and end, home application (no information how much) of reflexology not considered in analysis
Sikorskii (2020)	RCT	RoB Randomised Assignment: low Deviations from the intended interventions: high Missing outcome data: low Measurement of the outcome: high Selection of the reported result: low Overall Risk of Bias: high	PRO: Sample: Homogeneity between arms Methodical quality: Associations among responses to multiple symptoms within patients were accounted for, dummy variable for differentiation between potentially different effects on different symptoms, patient level covariate analysis CONTRA: Methodical quality: no blinding Report quality: no data on dropouts, no information on ethics committee approval, no specification of location and type of pain
Samancioglu (2019)	RCT	RoB Randomised Assignment: high Deviations from the intended interventions: some concern Missing outcome data: low Measurement of the outcome: high Selection of the reported result: low Overall Risk of Bias: high	PRO: Methodical quality: Active control group CONTRA: Sample: baseline differences between groups, small sample Methodical quality: no testing for normal distribution of data, per protocol analysis, no power analysis Report quality: no clear differentiation who dropped out of the study
Wyatt (2021)	RCT	RoB Randomised Assignment: low Deviations from the intended interventions: some concern Missing outcome data: low Measurement of the outcome: high Selection of the reported result: low Overall Risk of Bias: high	PRO: Sample: large sample, homogeneity between arms Methodical quality: patients with missing data points analysed due to LME model, inclusion of balancing factors for randomization, blinding of interviewers, Control group despite SMART, power analysis Report quality: Comparison of baseline values of dropouts, CONTRA: Sample: majority of sample are white women which are most interested in this kind of therapy according to research Methodical quality: bigger dropout for meditative practices (suitable protocol?) no correction for multiple testing, possibly varying frequency of intervention between patients, patients actively approached during hospital visits (possible bias), after week 4 high risk of bias due to differentiation between responders and non-responders Report quality: no information on other medication
Sikorskii (2018)	RCT	RoB Randomised Assignment: some concern Deviations from the intended interventions: low Missing outcome data: low Measurement of the outcome: high Selection of the reported result: low Overall Risk of Bias: high	PRO: Report quality: Effect sizes included CONTRA: Report quality: No differentiation between intervention and control group at baseline
Rambod (2019)	RCT	RoB Randomised Assignment: some concern Deviations from the intended interventions: low Missing outcome data: low Measurement of the outcome: high Selection of the reported result: low Overall Risk of Bias: high	PRO: Sample: No dropouts Methodical quality: Power analysis, intervention by certified reflexologist, blinding of outcome assessor, testing for normal distribution, ANCOVA for comparison between groups CONTRA: Methodical quality: no controlling for multiple testing Report quality: no information on other treatments
Nourmohammadi (2019)	RCT	RoB Randomised Assignment: some concern Deviations from the intended interventions: some concern Missing outcome data: low Measurement of the outcome: high Selection of the reported result: low Overall Risk of Bias:	PRO: Sample: Homogeneity between groups Methodical quality: Double blind, ANCOVA Report quality: information on belief in palliative care CONTRA: Methodical quality: hard to blind patients, no testing for normal distribution, randomization based on days of the week Report quality: No data directly after intervention periods, no information on other treatments and comorbidities, no information on who performed the intervention
Hesami (20,219)	RCT	RoB Randomised Assignment: some concern Deviations from the intended interventions: low Missing outcome data: low Measurement of the outcome: high Selection of the reported result: low Overall Risk of Bias: high	PRO: Sample: Homogeneity among groups Methodical quality: power analysis, ANCOVA CONTRA: Methodical quality: short study period, researcher applied intervention, no testing for normal distribution, no follow up Report quality: no detailed information on other treatments, no information on blinding
Dikmen (2019)	RCT	RoB Randomised Assignment: some concern Deviations from the intended interventions: high Missing outcome data: high Measurement of the outcome: high Selection of the reported result: low Overall Risk of Bias: high	PRO: Sample: Homogeneity between groups Methodical quality: Accounting for washout time of analgesic medications before intervention, power analysis, testing for normal distribution, ANOVA CONTRA: Methodical quality: randomization by researcher, intervention applied by researcher, researcher blinded for analysis (can he really be blind if he knew the allocation before?), blinding of patients not really possible, shorter sessions for progressive muscle relaxation, no controlling for multiple testing, effects of analgesics may last longer than the 30 and 60 min used in the study, dropout of patients because they didn’t match inclusion criteria (this could have been sorted out earlier) Report quality: few baseline information, full results only presented visually and not numerically (only p-values), no data on analgesic use, number of patients randomized is much bigger than number of patients participating (what happened?)
Rezaei (2021)	RCT	RoB Randomised Assignment: some concern Deviations from the intended interventions: some concern Missing outcome data: some concern Measurement of the outcome: low Selection of the reported result: some concern Overall Risk of Bias: some concern	PRO: Sample: Methodical quality: power analysis, testing for normal distribution, Report quality: study registered in Iranian clinicaltrials.com registry CONTRA: Sample: Methodical quality: first author applied reflexology (not double blind), blinding of patients is not possible, researcher had direct contact with patients (high risk of bias), no controlling for multiple testing, very short time frame (one day) Report quality: no clear information if researcher was actually a trained reflexologist, process unclear (who evaluated the data?)
Jahani (2018)	RCT	RoB Randomised Assignment: some concern Deviations from the intended interventions: high Missing outcome data: high Measurement of the outcome: high Selection of the reported result: low Overall Risk of Bias: high	PRO: Sample: Homogeneity between groups, power analysis CONTRA: Methodical quality: blinding is not really possible, no testing for normal distribution, no controlling for multiple testing Report quality: No information on other treatments or comorbidities, no information on dropouts, no further details on control group (probably only standard care then), process is not entirely clear from the text
Kurt (2018)	RCT	RoB Randomised Assignment: some concern Deviations from the intended interventions: high Missing outcome data: high Measurement of the outcome: high Selection of the reported result: low Overall Risk of Bias: high	PRO: Methodical quality: power analysis Report quality: information on chemotherapy CONTRA: Methodical quality: difference in number of patients in arms, no blinding, big dropout (problems with study design or recruiting?) Report quality: some patients apparently didn’t want to answer some questions precisely
Dyer (2013)	RCT	RoB Randomised Assignment: some concern Deviations from the intended interventions: low Missing outcome data: low Measurement of the outcome: high Selection of the reported result: low Overall Risk of Bias: high	PRO: Sample: homogeneity between groups, low drop out Methodical quality: power analysis, Intention-to-treat analysis for primary outcome CONTRA: Methodical quality: no blinding, per protocol analysis for other outcome Report quality: outcome data for all only for primary outcome
Hodgson (2012)	RCT	RoB Randomised Assignment: some concern Deviations from the intended interventions: low Missing outcome data: low Measurement of the outcome: low Selection of the reported result: low Overall Risk of Bias: some concern	PRO: Methodical quality: active control group, crossover design with washout CONTRA: Sample: homogenous sample Methodical quality: no blinding Report quality: statistical analysis incomprehensible
Özdelikara (2017)	RCT	RoB Randomised Assignment: some concern Deviations from the intended interventions: high Missing outcome data: high Measurement of the outcome: high Selection of the reported result: low Overall Risk of Bias: high	CONTRA: Methodical quality: no control for multiple testing Report quality: no information on dropouts
Özdelikara (2017)	RCT	RoB Randomised Assignment: some concern Deviations from the intended interventions: high Missing outcome data: high Measurement of the outcome: high Selection of the reported result: low Overall Risk of Bias: high	CONTRA: Methodical quality: no control for multiple testing Report quality: no information on dropouts
Sharp (2010)	RCT	RoB Randomised Assignment: low Deviations from the intended interventions: low Missing outcome data: low Measurement of the outcome: high Selection of the reported result: low Overall Risk of Bias: high	PRO: Sample: large sample size, homogeneity between groups Methodical quality: active control, Intention-to-treat analysis, control for multiple testing CONTRA: Report quality: one sided interpretation of results, risk for reporting bias
Stephenson (2007)	RCT	RoB Randomised Assignment: some concern Deviations from the intended interventions: some concern Missing outcome data: low Measurement of the outcome: high Selection of the reported result: high Overall Risk of Bias: high	PRO: Sample: larger sample size, homogeneity between groups Methodical quality: statistical analysis CONTRA: Methodical quality: active but not completely comparable comparison group Report quality: reporting bias (only one of two scales for pain reported)
Stephenson (2000)	RCT	RoB Randomised Assignment: some concern Deviations from the intended interventions: high Missing outcome data: high Measurement of the outcome: high Selection of the reported result: some concern Overall Risk of Bias: high	CONTRA: Sample: small sample size Methodical quality: incorrect statistical analysis for crossover design, for all except one outcome only part of the sample was analysed
Uysal (2017)	RCT	RoB Randomised Assignment: some concern Deviations from the intended interventions: high Missing outcome data: high Measurement of the outcome: high Selection of the reported result: high Overall Risk of Bias: high	PRO: Sample: active control Methodical quality: control for multiple testing, power analysis CONTRA: Sample: baseline differences, differences in groups regarding tumour grade Methodical quality: no blinding, differences in length of sessions between interventions Report quality: reporting bias
Wyatt (2012)	RCT	RoB Randomised Assignment: low Deviations from the intended interventions: low Missing outcome data: low Measurement of the outcome: high Selection of the reported result: low Overall Risk of Bias: high	PRO: Sample: large and multicentric sample Methodical quality: Intention-to-treat analysis CONTRA: Sample: Methodical quality: no blinding Report quality: no concrete results for comparison of active groups
Tsay (2008)	RCT	RoB Randomised Assignment: some concern Deviations from the intended interventions: some concern Missing outcome data: low Measurement of the outcome: high Selection of the reported result: high Overall Risk of Bias: high	PRO: Sample: homogeneity between groups Methodical quality: power analysis CONTRA: Methodical quality: termed as double blind but no blinding possible, amount of narcotic analgesia consumption not evaluable Report quality: no information on adverse effects or conflict of interest
Wyatt (2017)	RCT	RoB Randomised Assignment: some concern Deviations from the intended interventions: low Missing outcome data: low Measurement of the outcome: high Selection of the reported result: low Overall Risk of Bias: high	PRO: Sample: large and multicentric sample Methodical quality: homogeneity between groups, Intention-to-treat analysis CONTRA: Sample: large dropout Report quality: no concrete information on activity in control group
Anderson (2021)	RCT	RoB Randomised Assignment: some concern Deviations from the intended interventions: low Missing outcome data: low Measurement of the outcome: high Selection of the reported result: low Overall Risk of Bias: high	PRO: Methodical quality: power analysis, reflexologist blinded so presession survey until after session CONTRA: Sample: small sample, no information on homogeneity Methodical quality: no direct comparison between groups, no comparison, no testing for normal distribution Report quality: no information on comorbidities
Ross (2002)	RCT	RoB Randomised Assignment: some concern Deviations from the intended interventions: high Missing outcome data: low Measurement of the outcome: high Selection of the reported result: low Overall Risk of Bias: high	PRO: Methodical quality: patients and interviewers blinded, correction for difference in group size, active control CONTRA: Sample: small sample Report quality: no information on data analysis
Hodgson (2000)	RCT	RoB Randomised Assignment: some concern Deviations from the intended interventions: low Missing outcome data: low Measurement of the outcome: high Selection of the reported result: low Overall Risk of Bias: high	PRO: Methodical quality: single blind CONTRA: Sample: no information on homogeneity and cancer type, small sample Methodical quality: no testing for normal distribution Report quality: differences regarding timing of post intervention questionnaire in the beginning, due to printing error some items were left out of the questionnaire, no data on patients pre intervention, not all p-values disclosed, no information on other therapies

## Efficacy of reflexology

### Pain

#### Description of studies

Fifteen RCTs dealt with the effects of reflexology on pain. In eight of these [5–13], the intervention was carried out by a certified reflexologist, in four [13–16], the intervention was carried out by caregivers who were taught how to apply the intervention and in three [17–19], the intervention was carried out by the researcher but further information on his qualifications regarding reflexology is missing.

Seven of the studies used an active control group [7, 9, 13–15, 17, 18], while the remaining seven used a passive one [5, 6, 8, 10–12, 16, 19].

Samancioglu Baglama et al. [15] included 64 patients with mainly hematologic disorders who received either a 60 min reflexology or reading session for 15 days. On the last day of intervention, the reflexology group showed a significantly better result on the VAS than the reading group (4.70 ± 1.55 vs. 6.36 ± 0.99; *p* = 0.000). In a study by Rambod et al. [5], the intervention was applied over five days and showed significant differences between intervention (2.72 ± 2.30) and control (4.33 ± 3.54;) at the end of the study (*n* = 72; *p* = 0.01). Dikmen et al. [18] analyzed 80 patients and already found significant differences for pain severity between groups at baseline (*p* = 0.001). Significant differences were also found at 3^rd^, 8^th^ and at 12^th^ week (follow-up) for pain severity and effect on daily life (*p*’s < 0.017) with the lowest scores found in the reflexology plus relaxation group. Jahani et al. [19] included 84 patients and collected data three days before and three days after a three day intervention, showing a significant group difference, with less pain in the intervention group already one day before the intervention (4.12 ± 2.18 vs. 6.57 ± 2.08; *p* = 0.001), as well as at day one (no p-value), two (*p* = 0.001) and three (*p* = 0.001) after. In a study by Stephenson et al. [14] data was collected from 90 patients before and after a reflexology session measuring pain with the brief pain inventory and the Short Form-McGill Pain Questionnaire (SF-MPQ). A significant difference between groups was found (*p* = 0.001), showing a bigger mean decrease in score in the intervention (1.1 points) compared to the control group (0.1 points; = 0.12). A subgroup analysis only analyzing the 32 patients with a score > 5 also showed a significant decrease in the intervention group with the decrease in score being even bigger (2.7 points) in the intervention group while the control group only decreased by 0.5 points (*p* = 0.007, = 0.23). Stephenson et al. [11] published another study, which included 36 patients and the intervention group received two sessions of reflexology 24 h apart. The authors found significantly less pain directly after the intervention in the reflexology group compared to the control group, which received standard care (*p* < 0.01). However, no such differences were found at three and 24 h after the intervention. Tsay et al. [8] investigated pain in 62 subjects with a VAS and the SF-MPQ applying reflexology on day two to four post-surgery for digestive cancer. Using the VAS, the authors found significantly lower values in the intervention group (β_G_ = -21.22 (4.93, *p* < 0.001) on average over all measurement points. Change of pain over time was also significantly different (*p* = 0.0107) with pain by trend staying the same in the intervention group while it was getting worse in the control group (β_I_ = -2.41 (1.38)), which also underwent surgery but received only standard care. For the SF-MPQ data were only collected at baseline and follow up at day five and six post-surgery and did not show any significant differences between groups but a decrease in pain in both arms, which over time was significantly stronger in the intervention group (β_I_ = -3.17 (1.41); *p *= 0.02). In a study with 40 patients by Anderson et al. [10] patients received one single session, showing a significant improvement on VAS scores from pre [mean = 4, 95% CI = 2.9, 5] to post session [mean = 1.6, 95% CI = 0.9, 2.2] for the intervention group (*p* < 0.0001) but not for the control group (mean = 3.7 pre and post session) which filled out surveys during a session. However, they did not directly compare the groups for outcomes but compared them regarding time since last pain medication showing no significant differences. Sikorskii et al. [16] in a secondary analysis of a study by Wyatt et al. [20] compared reflexology to a control group which only received calls for symptom assessment. They compared the Patient Reported Outcomes Measurement System (PROMIS) and Legacy measures (a group of questionnaires) for various outcomes at baseline and one week after the intervention. Significant differences between intervention and control were found one week after the intervention for both PROMIS-29 pain severity profile v1.0 (*p* = 0.04, ES = 0.31) and M.D. Anderson symptom inventory (MDASI) pain severity (*p* < 0.01, ES = 0.46) with better results in the intervention group. Stephenson et al. [12] investigated pain in 24 patients with breast and lung cancer using a crossover trial. One group received one reflexology session (30 min) and three days of no intervention with a 30 min control session on the last day and the opposite way for the other group. Measurements were taken before and after the first and the last session using three scales. The SF-MPQ showed significantly better results (mean difference = -0.41; *p* < 0.05) after reflexology compared to after the control session, while the SF-MPQ:PPI (present pain intensity) Scale and the VAS, both measuring pain intensity, did not find such differences. For all three scales only patients with breast cancer were included. Uysal et al. [13] who included 65 patients for five weeks (two interventions weekly) investigated adverse effects and found significantly less pain with grade 2 + in the reflexology group comparing it with control in week four (*p* = 0.002) and five (*p* < 0.001).

Four studies did not report any significant differences after six weeks with the Bayly Method [17] or subscales of the brief pain inventory [6], of which Wyatt et al. [9] used the pain intensity subscale in their cross-over trial (reflexology and Swedish massage, four weeks, washout one week). Hodgson et al. [7] also did not find any significant differences between the two study groups for any time point using the checklist of nonverbal pain indicators (CNPI).

Four of the eight studies in which a certified reflexologist applied the intervention showed significant results in favor of the intervention [5, 8, 11, 12], all three in which a caregiver applied the intervention [14–16] and two of the three with missing information [18, 19].

#### Methodical assessment of studies:

In the study by Dikmen et al. [18], the authors only reported p-values and presented results graphically without providing further information, making an interpretation in terms of clinical significance very difficult. Additionally, the enrollment and allocation process are difficult to understand with a huge dropout and no sufficient baseline information exist. Full blinding of the researcher for statistical analysis as stated in the study is impossible as the researcher conducted the allocation. Furthermore, in the study it wasn’t accounted for the same session duration of all interventions. Stephenson et al. [11] and Sikorskii et al. [16] did not provide information on the homogeneity of the groups or lack thereof [17]. Two other studies are either lacking information on dropouts [19] or had a huge dropout [6]. In the study by Tsay et al. [8], there might be an interference of analgesics with the intervention and one study by Stephenson et al. [14] shows risk of a reporting bias as pain was measured with two tools while reporting only one of them without clarifying which one. Stephenson et al. [12] formed mean values means of both groups and not within group, so patients were not their own control anymore in this crossover trial. This incorrect analysis doesn’t allow for interpretation of the results. This applies to two other studies, as well, as the statistical analysis is incomprehensible [7] or only intragroup comparisons were made [10]. The study by Wyatt et al. [9] also shows a risk for sampling bias and reduced reporting as no results were reported comparing the two active groups except for dyspnoe, demonstrating a significant result.

### Anxiety and depression

#### Description of studies

In eight of the studies dealing with anxiety and depression the intervention was delivered by certified reflexologists [8, 9, 12, 13, 21–24] while in five it was caregivers delivering it [14–16, 25, 26]. In three more studies the researchers applied reflexology but no information are given regarding their qualifications [17, 19, 27].

Eight of the studies used an active control group [9, 13–15, 17, 22, 23, 25], whereas the other eight used a passive one [8, 12, 16, 19, 21, 24, 26, 27].

Eight RCTs found a significant effect of reflexology on anxiety and depression in cancer patients [12–17, 19, 27]. Mantoudi et al. [17] reported a significant difference in change between the reflexology and relaxation group when comparing baseline values with 4^th^ (*p* = 0.006, η^2^ = 0.094) and 6^th^ week (*p* = 0.001, η^2^ = 0.138) for depression. For anxiety, however, no significant difference in change was found. Göral Türkcü et al. [27] applied reflexology to 62 patients with gynecological cancers over two weeks and found an advantage for the reflexology group two weeks after the end of the intervention for anxiety (*p* < 0.001) and depression (*p* < 0.001). Samancioglu Baglama et al. [15] and Stephenson et al. [14] both used a VAS to explore the effects of reflexology on anxiety. Both found significant differences, in favor of the reflexology group at day 15 (*p* = 0.036) and directly after a one time intervention (*p* = 0.001, ε^2 = 0.13), respectively. The latter also did a subgroup analysis for patients with anxiety > 5 revealing a significant difference (*p* = 0.006; = 0.15). In another study by Stephenson et al. [12] significantly better results were observed for anxiety after a reflexology compared to a control session (mean difference = -21.83; *p* < 0.000). This time, both, breast and lung cancer patients were analyzed. Using the Spielberger State-Trait Anxiety Inventory, Jahani et al. [19] found a significant advantage of the reflexology group on day three after treatment (n = 84; p = 0.04;), while Rezaei et al. [24] did not find a significant difference (n = 74). Still, there are differences that need to be considered. Rezaei et al. [24] merely did a before and after comparison taking place on the same day whereas Jahani et al. [19] had a three day intervention period and collected data only on day three after the intervention period. Furthermore, they had a passive control group whereas Rezaei et al. [24] had a researcher stand at bedside of the control group and no further information are given about their contact. Sikorskii et al. [16] found significant differences in favor of reflexology compared to a control group for depression using the Center for Epidemiologic Studies Depression Scale (short: CES-D; ES = 0.32, p = 0.03), MDASI distress severity (ES = 0.31 and p = 0.04) and SF-36 mental health (ES = 0.51,* p* < 0.01). Using PROMIS-29 depression profile v1.0 and MDASI sadness severity, no significant differences were observed. For anxiety significant differences were observed using the PROMIS-29 anxiety profile v1.0 (ES = 0.30, p = 0.04) and the Spielberger State-Trait Anxiety Scale (ES = 0.39, *p* = 0.01). Though, Wyatt et al. [9] also used this scale but could not find any significant differences comparing reflexology, foot massage and a control group.

Eight other studies also did not find a significant difference comparing groups [8, 9, 21–26].

Wyatt et al. [25] conducted a sequential multiple assignment trial comparing reflexology to meditative practices, also including a control group. After 4 weeks nonresponding patients were randomized 1:1 to either the same group or the other group, while responsive patients continued their treatment for another four weeks. No significant results were reported for anxiety and depression. Tsay et al. [8] (*n* = 62, day 2–6 after surgery) did not find any significant differences for anxiety on average over all measurement points but a decrease in symptoms which was significantly stronger in the intervention group (β_I_ = -1.12 (0.49); p = 0.0231). This also applies to a study by Murat-Ringot et al. [21] (*n* = 80) in which reflexology was compared with a control group. Patients received four sessions of reflexology (30 min each) every two to three weeks during chemotherapy infusion depending on the chemotherapy protocol for four cycles. In a study by Rezaei et al. [24] (*n *= 74) patients received two sessions in one day but no significant differences were observed after the intervention compared to a control group. Sharp et al. [22] (*n* = 183, reflexology + SIS, scalp massage + SIS, self-initiated support for eight weeks), Ross et al. [23] (*n* = 26, reflexology, foot massage for six weeks), Sikorskii et al. [26] (*n* = 256, reflexology and control for four weeks) and Wyatt et al. [9] (*n* = 286, reflexology, foot massage, control for four weeks) did not find any significant results, as well.

Only one of the seven studies in which a certified reflexologist applied the intervention showed significant results in favor of the intervention [12] and only two out of five when it was applied by a caregiver [15, 16]. This is also the case for all three studies, in which no detailed information are given [17, 19, 27].

#### Methodical assessment of studies

In three studies [17, 19, 27], no information is given on other treatments, medication and comorbidities. Göral Türkcü et al. [27] also did not provide information on the control group but only on the intervention group after the second and final week of intervention which results in risk for reporting bias regarding the short term effect. Three other studies [12, 15, 16] display some methodical drawbacks. Stating limitations of their study, Sikorskii et al. [16] noted that there are methodological drawbacks so the results are not reliable for depression. Samancioglu Baglama et al. [15] did not test for normal distribution of data while the study design of the study by Stephenson et al. [12] doesn’t allow for interpretation of results. Murat-Ringot et al. [21] allowed home application of reflexology but did not consider it in their analysis. Consequently, not all data necessary for interpretation of the results is available. Sikorskii et al. [26] did not provide information on drop outs at all and Ross et al. [23] had a drop out of a third which was possibly caused by foot discomfort as this was noted as a common side effect. The latter, additionally, had a small sample size (*n* = 26) to begin with. In the study by Rezaei et al. [24], it is not clear who evaluated the data, so it cannot be ruled out that the reflexologist was involved here. Furthermore, the researcher stood at the patients’ bedside in the control group, with no information on possible verbal interaction between them. Due to the attention patients might have received hereby, this passive control group could possibly be considered as an active control. Wyatt et al. [25] randomized patients a second time depending on their outcomes after the first four weeks. Since this is not a complete randomization anymore and includes a high risk of bias, we only considered results of the first randomization. Furthermore, patients might have received varying frequencies of intervention making it hard to compare.

### Fatigue

#### Description of studies

With fatigue being a very common side effect in cancer patients, nine studies investigated whether reflexology could be a useful tool in alleviating these symptoms. In four of the studies investigating the effects on fatigue reflexology was delivered by certified reflexologists [5, 9, 13, 28, 29] in three by caregivers [15, 16, 25] and in two it either isn’t clear who performed the intervention [30] or if the researcher applying it had any qualifications for doing so [18].

Four studies used an active control group [9, 13, 15, 18, 25] while the other five used a passive one [5, 16, 28–30].

The study by Rambod et al. [5], which included only patients with Hodgkin- and Non-Hodgkin Lymphoma, used the Multidimensional Fatigue Inventory and found significant differences between groups in favor of reflexology after the five day intervention period (*p* < 0.001). Significant differences after the intervention were also found for four of the five subdimensions of the inventory: general fatigue (p = 0.006), physical fatigue (*p *= 0.01), reduced activity (*p* = 0.01) and reduced motivation (*p* = 0.05). Nourmohammadi et al. [30] included 60 patients and obtained significant results in favor of reflexology between groups two months after the end of the four week intervention period (*p* = 0.000), showing possible long-term effects of the intervention. Conducting a pre-to-post comparison, Hesami et al. [28] included 80 subjects and, also using the Fatigue Severity Scale, found a significant difference between groups (*p* = 0.016) with less fatigue in the reflexology group. In the study by Dikmen et al. [18], the authors reported significant differences between groups at the 3^rd^, 8^th^ and 12^th^ (follow up) week for both fatigue severity (3rd: *p* = 0.001; 8^th^: *p* = 0.001; 12^th^: *p *= 0.039) and effects of fatigue on daily life (all *p*-values = 0.001) with the lowest scores being reported in week eight for reflexology plus progressive muscle relaxation. Özdelikara et al. [29], who included 60 patients, observed significant differences between groups for fatigue severity (*p* < 0.05) and daily life activity exposure levels (*p* = 0.05) after the fourth chemotherapy treatment cycle. While investigating adverse effects, Uysal et al. [13] found that the reflexology group presented with significantly less grade 2 + fatigue when compared to the foot massage and control group in week 3 (*p* = 0.03), 4 (*p* < 0.001) and 5 (*p* = 0.036). Sikorskii et al. [16] used three different measurements to assess fatigue, only finding significant differences between groups using the MDASI fatigue severity scale (*p* = 0.03). Two other studies did not find any significant differences [9, 25]. Samancioglu Baglama et al. [15] found no significant differences during and after the intervention but already at baseline, showing more fatigue in the intervention group (*p* = 0.01).

Three of the four studies in which a certified reflexologist applied the intervention showed significant results in favor of the intervention [5, 28, 29], only one of the three in which a caregiver applied it [16] and both when no detailed information exist on this matter [18, 30].

#### Methodical assessment of studies

Four studies show some methodical problems [15, 16, 29, 30]. Sikorskii et al. [16] did not provide information on significance for baseline differences between groups whereas in the study by Nourmohammadi et al. [30], randomization was done based on days of the week leading to a high risk of bias. They also gathered information on patients’ believe in the impact of palliative practices showing that it was 20% higher in the reflexology than in the control group. This might have created a placebo effect since blinding factually couldn’t be achieved. Özdelikara et al. [29] did not control for multiple testing and there is also a potential risk for performance bias since patients were lying on ergonomic beds during reflexology sessions and there is no information on whether the control group was allowed to use these beds as well at some point. Baseline differences for fatigue in the study by Samancioglu Baglama et al. [15] put both the randomization and the validity of the result into question.

### Quality of life / symptom severity and distress

#### Description of studies

In eight of the studies investigating Quality of Life or Symptom Severity and Distress the intervention was applied by a certified reflexologist [6, 9, 13, 21–23, 31, 32]. Three studies had caregivers apply the intervention [20, 25, 26] while in four studies no information are given on whether the researcher had any qualifications regarding reflexology [17, 18, 27, 33].

Eight studies used active control groups [9, 13, 17, 18, 22, 23, 25, 33], while the other six used passive groups [6, 20, 21, 26, 27, 31].

Quality of life, physical and social functioning and symptom distress and severity are all composed or representative of multiple symptoms, therefore showing a broader picture of the condition of the patients. Mantoudi et al. [17] examined the difference between a reflexology and a relaxation group for QoL over six weeks and found significant differences in change from baseline to after six weeks for the mental component summary score (*p* = 0.017, η^2^ = 0.071) and the physical component summary score (*p* < 0.01, η^2^ = 0.168). In a study by Göral Türkcü et al. [27] the global quality of life scale showed a significant difference between groups two weeks after the intervention [intervention: mean = 60.22 (SD = 17.17), control (mean = 40.59 (SD = 9.06), *p* < 0.01)] which was also found for the functional scale (*p* < 0.001) and symptom scale (*p* < 0.001). A study by Hodgson et al. [32], which included 12 people, compared reflexology with placebo reflexology on day one, three and five of their stay in the hospital. They reported a significant difference for the subcomponent of breathing (*p* = 0.026) and overall (*p* = 0.004). Dikmen et al. [18] also reported significant results for the 3^rd^, 8^th^ and 12^th^ week (follow up) (*p* < 0.05), with the highest scores of quality of life being reported in the 8^th^ week [mean = 6.11 (SD = 0.274)] in the group receiving reflexology plus progressive muscle relaxation. No significant results for reflexology were found in a study by Sharp et al. [22] using FACT-B: TOI at 18 weeks post surgery but 24 weeks post surgery where reflexology plus self-initiated (SIS) support lead to a better outcome than SIS alone (*p* = 0.02) but did not show a significant difference when compared to the scalp massage plus SIS group. Using the Functional Assessment of Cancer Therapy-breast cancer version (FACT-B) total score a significant difference between the intervention and SIS group was detected at 24 weeks post surgery, as well (*p* = 0.03). A study by Kurt et al. [6] using the European Organization for the Research and Treatment of Cancer Quality of Life Questionnaire Chemotherapy-Induced Peripheral Neuropathy (EORTC QLQ-CIPN-20) only found a significant difference between intervention and control group in the last week of the six-week intervention period for the sensory function subscale (*p* = 0.024) while the other two subscales did not show any significant results. Uysal et al. [13] investigated adverse effects and found significantly less pain and fatigue (see above for detailed results). Furthermore, significantly lower grade 1 + urinary frequency in week 5 (*p* = 0.044) and grade + 1 distension in weeks 4 (*p* < 0.000) and 5 (*p* < 0.000) were found for the reflexology group compared to control. This study and two other studies [21, 31] also measured quality of life using the EORTC-Quality of Life Questionnaires (QLQ)-C30. Uysal et al. [13] found significantly better results on the function scale for the reflexology group compared to control for week 3 (*p* < 0.000) and 5 (*p* < 0.000). On the symptom scale significantly better results were found in the reflexology group compared to control (*p* = 0.003) while the reverse was found in week 5 (*p* < 0.000). Comparing groups for the global health scale, significant differences in favor of reflexology compared to both other groups were detected in week one (*p* = 0.012), three (p < 0.000) and five (*p* < 0.000). Özdelikara et al. [31] too examined the QoL and significant differences between groups for posttest measurements (24h after last chemotherapy cycle) for the general health score (*p* = 0.000), function score (*p *= 0.000) and symptom score (*p* = 0.000) were detected. Murat-Ringot et al. [21] did not find any significant differences. Wyatt et al. [9], using QoL FACT-B in their study also did not detect any diversity between the reflexology and the foot massage or control group when examining quality of life, as well as another study by Wyatt et al. [20], which used the Quality of Life Index and included 256 patients, where reflexology was compared with attention by the caregiver. Patients received at least one caregiver delivered reflexology session per week (real average 1.1) for the first four weeks. After that, there was no requirements and patients reported an average of 0.6 sessions per week until follow up in week 11. This study [20] also found significantly better results for the intervention group for summed symptom severity using MDASI and an adjusted coefficient of group variables over week five and eleven (p = 0.02) and significantly better results in week two (*p* < 0.01), 3 (*p* = 0.01) and five (*p* < 0.01) which can mainly be attributed to improvements in pain and fatigue. Sikorskii et al. [26], including 209 patients, also investigated symptom severity in an additional secondary analysis of this study by Wyatt et al. [20] only taking into account the first four weeks. Significantly better results in favor of the reflexology group were found for pain (*p* = 0.03) with no significant results in any of the other domains. Ross et al. [23] did not find any significant results looking at symptom distress except reportedly significantly greater appetite and mobility in the foot massage group, but no p-values were given. Dyer et al. [33] included 115 patients, who received four sessions of either aromatherapy or reflexology over the course of ten weeks on average. Results of the first concerns score of the Measure Yourself Concerns and Wellbeing (MYCaW) show a significant difference in favor of aromatherapy (*p* = 0.046) while the second concerns score shows no significant difference between groups but a significant improvement within groups (no p-values for comparison). This is also the case for overall wellbeing scores (no p-values for comparison). The study by Wyatt et al. [25] also found no significant differences between groups for symptom severity.

Four of the six studies in which a certified reflexologist applied the intervention showed significant results in favor of the intervention [6, 13, 22, 31], two of three when caregivers applied it [20, 26] and three of four when no detailed information exist [17, 18, 27].

#### Methodical assessment of studies

In the study by Uysal et al. [13], not only did the reflexology group receive longer sessions than the foot massage group but there were also significant differences in global QoL between groups at baseline. Furthermore, the control group was comprised of much more patients with grade III compared to grade II tumor than the reflexology group, which could possibly have influenced the patients’ general wellbeing. Finally, the authors did not provide information on results of EORTC QLQ CR29 as planned, resulting in a reporting bias. Wyatt at el. [20] investigated both symptom severity and QoL. However, since the authors did not describe what attention by the caregiver in the control group as an intervention looked like, it is hard to draw a deduction for the actual efficacy. In the study by Hodgson et al. [32], no consistent timing of the post intervention questionnaire existed in the beginning, according to the authors items were left out from the questionnaire due to printing errors and other information like homogeneity between group and p-values are missing. Dyer et al. [33] failed to present p-values for a group comparison for the secondary concern of the MYCaW score so no conclusion can be drawn from this outcome.

### Physical and social functioning / interference with daily life

In one of the three studies examining these endpoints the intervention was applied by a certified reflexologist [9] while the other two had caregivers apply it [16, 20].

One study used an active control group [9] and two studies used a passive one [16, 20].

Wyatt et al. [9] investigated physical functioning and discovered significantly better results in the reflexology group compared to control (*p* = 0.04) but found no significant differences between reflexology and foot massage. In addition, the effect on dyspnea was measured showing significantly better results for reflexology when compared to control (*p* < 0.01) and foot massage (*p* = 0.02). In another study, Wyatt et al. [20] found no significant differences between groups for physical functioning and satisfaction with participation in social roles, while they observed significantly better results for reflexology using an adjusted coefficient of group variables over week five and eleven (*p* < 0.01) and significantly better results in week two (*p* = 0.02), 3 (*p* < 0.01) and 5 (*p* < 0.01). Sikorskii et al. [16] found no significant differences between reflexology and a control group when comparing different PROMIS and legacy measures for physical functioning. Comparing groups for social functioning, however, significant differences in favor of reflexology were found using SF-36 social functioning (legacy) (*p* = 0.04), while PROMIS-29 satisfaction with participation in social roles showed insignificant results.

The only study [9] where reflexology was applied by a certified specialist showed significant results in favor of the intervention for physical functioning but not interference with daily life while results are very mixed for the studies in which caregivers applied it. Sikorskii et al. [16] and Wyatt et al. [20] each showed significant results in favor of the intervention for only one of the above outcomes.

#### Methodical assessment of studies

In the study by Wyatt et al. [9], there is a risk for sampling bias and they did not report results comparing the two active groups except for dyspnea. In another study by Wyatt et al. [20], no information are provided regarding details on the control group, which received attention by their caregivers. Sikorskii et al. [16] did not provide information on the homogeneity of groups.

### Nausea and vomiting

In all the studies investigating nausea and vomiting reflexology was applied by a certified reflexologist.

Only one study used an active control group [9] while the other three studies used a passive one [10, 21, 29].

Two studies investigated the effect of reflexology on chemotherapy induced nausea and vomiting at which only the study by Özdelikara et al. [29] found significant differences between groups using the Rhodes index of nausea, vomiting and retching which is composed of three subscales. A significant advantage for reflexology was found for the subscale of symptom development scale (*p* = 0.000) and distress development (*p* = 0.000) after the 4^th^ cycle of CTX, while none was found regarding if they experienced symptoms or not. Murat-Ringot et al. [21] measured CINV during the second cycle of chemotherapy, asking patients to fill out a VAS before and after the reflexology intervention or upon entering and leaving the hospital for the control group respectively. An intention-to-treat analysis was conducted, with patients with missing outcome data being considered as having an increase of > 2 on the VAS. While the per-protocol analysis showed an advantage for the intervention (*p* = 0.001), the intention-to treat did not find an effect. Two more studies also investigated nausea only, with Anderson et al. [10] (*n* = 40; one session with pre and post test), who used VAS and only looked at the in-group difference, and Wyatt et al. [9] who used the nausea item from the physical subscale of FACT-B, both not finding significant results.

#### Methodical assessment of studies

As mentioned above, the study by Wyatt et al. [9], shows risk for sampling bias and incomplete reporting. Murat-Ringot et al. [21] allowed home application of reflexology but did not consider it in their analysis. Therefore, data is missing for interpretation of the results in its entirety. In the study by Özdelikara et al. [29], it was not controlled for multiple testing and there might be a risk for performance bias, whereas Anderson et al. [10] did not provide information on homogeneity between groups.

### Sleep, mood and relaxation

In three studies the intervention was carried out by certified reflexologists [5, 7, 22], in one study caregivers applied it [16] and in one there are no information on the researchers qualifications who applied it [33].

Three studies used an active control group [7, 22, 33], while two used a passive control group [5, 16].

Rambod et al. [5] found significant differences between groups in favor of the intervention group for two of the scales when investigating sleep quality after five days of reflexology (subjective sleep quality, *p* < 0.001; sleep latency, *p* = 0.001). Total sleep quality at baseline already showed better values for the reflexology group (*p *= 0.05), though, suggesting a potential problem with randomization. After the intervention differences were still significant (*p* < 0.001). No significant differences were found in this study by Sikorskii et al. [16] comparing sleep disturbance using PROMIS-29 and MDASI (legacy). Hodgson et al. [7] compared groups for affect by observing patients four times per day on intervention day for four weeks for five minutes each time and then averaged measures for mean values. No significant differences between groups for both negative and positive mood were found. Sharp et al. [22] found significant differences between groups at 18 weeks after surgery in favor of reflexology (*p* < 0.0005) and scalp massage (*p* < 0.0005) compared to control and significant differences for reflexology compared to control at 24 weeks post surgery (*p* = 0.02) using the Mood Rating Scale (MRS) relaxation subscale. The easy-goingness subscale also revealed significant differences in favor of reflexology compared to scalp massage (*p* = 0.04) and control (*p* < 0.0005) at 18 weeks post surgery. Dyer et al. [33] compared pre and post session scores for relaxation between reflexology and aromatherapy groups for all four sessions and for change over all four sessions which on average were distributed over ten weeks but did not find significant differences for both.

Two of the three studies where reflexology was applied by a certified specialist showed significant results in favor of the intervention [5, 22], while no such benefits could be observed for all other studies.

#### Methodical assessment of studies

As mentioned above, the statistical analysis in the study by Hodgson et al. [7] is incomprehensible and no information was provided on homogeneity of groups [16].

### Narcotic analgesia consumption

In the study by Tsay et al. [8], the intervention was applied by a certified reflexologist a passive control group was used. It is the only one included which also investigated the influence of reflexology on narcotic analgesia consumption as an outcome providing reflexology on days two to four after cancer surgery. At follow up on day five and six after surgery for hepatocellular or gastric carcinoma the intervention group showed a significantly lower use in Demerol than the control group (p = 0.015). However, there might be an interference of analgesics with the intervention.

### Self Esteem / psychiatric morbidity

In both studies listed here the intervention was applied by a certified reflexologist.

One study used an active control group [22] and one used a passive one [21].

Murat-Ringot et al. [21] measured self-esteem and found no significant differences between groups at the end of the study. At the end of the study a Body Image Questionnaire was used while baseline values were collected using the Rosenberg self-esteem scale, which makes interpretation over the course of the study difficult. Furthermore, as mentioned above, home application of reflexology was not accounted for in the analysis. Sharp et al. [22] investigated psychiatric morbidity and found no significant differences between groups.

### Perceived social support / quality of relationship between caregiver and patient

The intervention was applied by caregivers in this study, which used a passive control group.

No significant differences between groups were found by Wyatt et al. [20] looking at perceived social support and the quality of the relationship between caregiver and patients. However, as noted previously, no information are provided regarding details on what the intervention in the control group looked like, who received attention by their caregivers.

#### Adverse events

No adverse events that can be attributed to reflexology were reported.

## Discussion

An overall problem in designing studies with an active intervention is that true blinding of patients is very hard to achieve, since patients are aware of the application of an intervention. A possibility to blind a patient is by applying a very similar technique to the same body part as done by a very small number of studies included. Still, there is no way to blind the people applying the intervention and/ or the control counterpart. Therefore, while often termed as single or even double blind, most studies included have an open design.

As pain is arguably one of the most relevant side effects cancer patients experience, 13 of the included studies investigated the effect of reflexology on pain. Nine of the studies showed at least partially significant results [5, 8, 11, 12, 14–16, 18, 19]. The study by Dikmen et al. [18] found that reflexology has a positive effect on pain. However, some strong methodical drawbacks such as incomplete reporting of information should be considered and the results should be viewed with caution. Other studies also lack information on homogeneity of groups [11, 16] or dropouts [19] whilst one study also shows risk for a reporting bias [14]. Incorrect analysis of the study by Stephenson et al. [12] doesn’t allow for interpretation of the results. Two of the studies showing significant advantages for reflexology [5, 11] only investigated the effect over a very short time (five days; directly and after 3h, 24h respectively), which might indicate an acute effect on pain. While five other studies’ results where insignificant [6, 7, 9, 10, 17] they also presented with similar problems.

All in all, due to some strong methodical drawbacks these findings should be viewed with caution and a clear conclusion cannot be deducted. As none of the studies investigating pain allows for true blinding, it is unclear if the positive effect is attributed to the intervention or a result of being relaxed, as the relaxation response might help alter pain perception [34].

As cancer patients get confronted with their diagnosis and the consequences, dealing with potential anxiety and depression is important. Seven studies reported significant effects of reflexology on anxiety and depression [12, 14–17, 19, 27]. Mantoudi et al. [17] only found a significant advantage for depression but not for anxiety after four and six weeks. More information on other treatments, medication and comorbidities would have been of value here due to the possibly multifactorial origin of anxiety and depression but no further information is given. The same lack of information also applies to Jahani et al. [19] and Göral Türkcü et al. [27] while the latter also show risk for reporting bias regarding the short term effect. Three other studies [13, 16{Sikorskii, 2018 #344]} showed significant results for anxiety. However, the study design in the study by Samancioglu Baglama et al. [15] doesn’t allow for data interpretation, while the study by Sikorskii et al. [16], which also showed partially significant results for depression, presented with measurement and statistical hypothesis testing errors and therefore unclear results.

Eight other studies [8, 9, 21–26] reported insignificant results. Murat-Ringot et al. [21] allowed home application of reflexology but did not consider it in their analysis, whereas other studies showed some methodical problems [23, 24, 26]. The study by Wyatt et al. [9] did not blind patients even though it would have been possible since the active control group received a foot massage.

Overall, these findings described on anxiety and depression do not hint to a benefit by reflexology. Studies that reported significant results in favor of reflexology are presented with some major drawbacks. Additionally, there was only one study which allowed for true blinding of patients [23]. The evidence supporting long term effects is very thin as only two of the studies [19, 27] investigating these effects showed significant improvements. This leaves the impression that reflexology can at best help in improving anxiety and depression in an acute scenario. This could possibly be explained by an increased level of relaxation, which wears off after returning to usual life with all its stressors.

Taking a closer look at the effects on fatigue, six out of nine studies showed significant results in favor of reflexology [5, 16, 18, 28–30]. Three of them show some methodical problems [16, 29, 30], such as lack of information on baseline comparisons [16], risk for placebo effect [30] and no control for multiple testing [29]. The results of two other studies [5, 28] indicate that reflexology might be a tool to mitigate chemotherapy-induced fatigue, especially physical fatigue, in the short term.

Three other studies [9, 15, 25] did not find significant results. In the study by Wyatt et al. [25], patients might have received varying frequencies of intervention making it hard to compare, while in the study by Samancioglu Baglama et al. [15], the control group already showed significantly less fatigue than the reflexology group at baseline. One might also argue that baseline differences in fatigue could also influence the perception of the two other parameters (pain and anxiety) examined in this study, which while showing significantly better results in the reflexology group, nonetheless, could have possibly shown even stronger effects.

To conclude, the above shortcomings need to be considered. Although the trend indicates that reflexology might have a positive impact on fatigue in cancer patients it remains unclear whether this also pertains to long term effects as only one of the above studies investigated possible long term effects but did not find significant results [9]. As fatigue in the context of cancer is a chronic state it is important to note that short term effects only offer a brief symptom relief. This implies that frequent application would be required for a meaningful impact. Furthermore, none of the studies investigating fatigue allow for true blinding of the patients. Since all the studies showing significant results except one [18] only had a passive control group, it is unclear whether other factors beyond the intervention might have played a role in the short term improvement.

Quality of Life is arguably the most comprehensive measure for cancer patients as it is more so a combined than a single outcome and therefore allows for covering more influencing factors.

10 studies presented with significant results in favor of reflexology [6, 13, 17, 18, 20, 22, 26, 27, 31, 32]. While the study by Mantoudi et al. [17] indicates that patients experience less restrictions of daily life due to physical limitations when using reflexology rather than simple relaxation, the study by Kurt et al. [6] suggests very little effect, as only a small part of the results was significant. Sharp et al. [22] found significantly better results compared to the control but not to the scalp massage group which implies that more than one form of physical intervention might result in the desired outcome. Three other studies come with some drawbacks [13, 20, 32] like differences in session length and risk for a reporting bias [13], lack of information on what attention by caregivers looked like [20] and missing information on p-values and more [31]. For this reason, the results of these three studies should be viewed with caution.

Five other studies did not produce significant results [9, 21, 23, 25, 33] and two of them present with methodical drawbacks [25, 33]. Dyer et al. [33] reported statistically and clinically relevant intragroup improvements for the aromatherapy and reflexology group but failed to present p-values for a group comparison for the secondary concern score. Due to this, no conclusion can be drawn from this outcome.

Overall, the results regarding QoL and symptom distress and severity are mixed. While more studies speak for a positive effect, some drawbacks limit the informative value. This again includes a lack of true blinding, which applies to only one study [23]. Therefore, the influence of a placebo effect should at least be considered.

The way patients can continue to navigate daily life is closely related to their QoL. In the study by Wyatt et al. [9], significant results in favor of reflexology were observed for physical functioning when comparing reflexology to a control group but not when compared to foot massage. Even though there was no blinding, this shows that reflexology is likely not better than other similar interventions for this outcome. However, reflexology might help with dyspnoe as examined in this study, as well. With Sikorskii et al. [16] showing a positive tendency for social but not physical functioning and Wyatt et al. [20] showing a significant improvement for interference with daily life but not physical functioning, the overall results are pretty ambiguous and don’t allow for a clear trend.

As it is a common side effect of chemotherapy, four studies [9, 10, 21, 29] also investigated whether reflexology might be a useful tool in alleviating nausea and vomiting. Three of them [10, 21, 29] investigated short term effects but only one [29] found at least partially significant results in favor of reflexology. However, Murat-Ringot et al. [21] showed that reflexology might potentially help reduce the dosage of antiemetic drugs needed to deal with delayed nausea and vomiting. Altogether, the trend points towards no significant efficacy of reflexology on nausea and vomiting, though.

Only a handful of studies reported on sleep, mood and relaxation [5, 7, 16, 22, 33]. Even though Sharp et al. [22] and Rambod et al. [5] found a significant effect, the overall results indicate that reflexology doesn’t seem to be superior to other interventions with the same goal.

While speaking for possible positive effects on the consumption of narcotic analgesics, only one study [8] investigated this outcome. Therefore, the evidence is too limited for a conclusion. This also applies to the outcomes of self esteem [21], psychiatric morbidity [22], perceived social support and quality of relationship between caregiver and patient [20], which all presented with insignificant results. Consequently, reflexology presumably is not an effective tool here.

Since no trend could be observed in terms of who applied the intervention, it likely does not make a difference. This once again raises the question, whether it is the intervention itself or simply the psychological and physical attention received by the patients, that has led to some significant benefits in favor of reflexology.

The studies included in this review investigated a variety of symptoms which we discussed mostly individually. However, it is important to note that changes in characteristics of one symptom and changes in the current state of disease might influence one another, as proposed in the biopsychosocial model [35]. Since no individual data on patients exists in the included studies, exploring such interactions for the most part is beyond this review.

### Limitations of this work

This review has a few limitations. All studies exclusively included adults which doesn’t allow for conclusions regarding children. Furthermore, only studies in German or English as well as only Randomized Controlled Trials were included, excluding grey literature. Something else to consider is that most studies show a high risk of bias with a small number showing a moderate risk. Additionally, some outcomes were only investigated by a small number of studies.

## Conclusion

Studies on reflexology included a wide variety of different types of cancer not restricting conclusions to a small group of cancer types. The reported results are very heterogenous. Most studies indicate that reflexology is superior to a passive control group for pain, quality of life and fatigue but not anxiety and depression. For other outcomes, the sample of studies is too small for a conclusion. As results are very mixed, no trend in efficacy could be observed looking at whether reflexology was performed by a certified professional or a caregiver. The methodical quality of the majority of studies is too poor for them to demonstrate proof for the specific efficacy of reflexology. Meanwhile, it appears that reflexology is not superior to other massage interventions as there exists no physiological concept on how these reflex zones work. Reflexology rather seems to draw its efficacy from the care and attention received through the intervention. This, in fact, can be achieved by any form of massage.

For future randomized controlled trials on reflexology in oncological treatment we would therefore like to recommend a few criteria to avoid possible bias. Control groups should be active with an intervention that’s as indistinguishable as possible from reflexology for the patient such as foot massage. This would allow for real blinding of patients. While a bit more extensive, possible subanalyses of patients who believed in such interventions prior to the trial and patients who did not could help shed more light on possible influencing factors. This could also be applied for other factors that are considered part of the biopsychosocial model, such as stress unrelated to the disease and patients’ support networks. Additionally, a protocol where all patients receive the intervention in the same time interval regarding their cancer therapy might be beneficial. This could help reduce the impact differences in time intervals between reflexology and cancer treatment might have on symptoms.

### Supplementary Information

**Additional file 1:** Table XX Excluded studies

## Data Availability

All data generated or analysed during this study are included in this published article (and its supplementary information files).

## Abbreviations

CINV: Chemotherapy-induced nausea
CNPI: Checklist of nonverbal pain indicators and vomiting
CTX: Chemotherapy
EORTC QLQ: European Organization for the Research and Treatment of Cancer Quality of Life Questionnaire
FACT-B: Functional assessment of cancer therapy Breast
MDASI: M.D. Anderson symptom inventory
MRS: Mood rating scale
MYCaW: Measure Yourself Concerns and Wellbeing
PROMIS: Patient Reported Outcomes Measurement System
QoL: Quality of life
SF-MPQ: Short form-McGill pain questionnaire
SIS: Self-initiated support
VAS: Visual analogue scale
