# Supplementary material for: Reflexology in oncological treatment – a systematic review
Source: BMC Complement Med Ther. 2024 Jan 11;24:32. doi: 10.1186/s12906-023-04220-4 (PMC10782728; doi:10.1186/s12906-023-04220-4)
Supplement: Supplementary file 1 — Additional file 1: Table XX Excluded studies [file 12906_2023_4220_MOESM1_ESM.docx]

## Table XX Excluded studies

| References | Study Type | Year | Title | Reason for exclusion |
| --- | --- | --- | --- | --- |

| Luo et al. [52] | RCT | 2019 | Healthcare service utilization and work-related productivity in reflexology intervention for advanced breast cancer women | Outcome not relevant |
| --- | --- | --- | --- | --- |
| Toygar et al. [52] | RCT | 2020 | Effect of reflexology on anxiety and sleep of informal cancer caregiver: Randomized controlled trial | Patients are caregivers and not cancer patients |
| Gok Metin et al. [52] | RCT | 2018 | Mind-Body Interventions for Individuals With Heart Failure: A Systematic Review of Randomized Trials | Patients are not cancer patients |
| Zengin et al.[52] | RCT | 2019 | The effects of sleep hygiene education and reflexology on sleep quality and fatigue in patients receiving chemotherapy | Multiple Interventions |
| Blackburn et al.[52] | RCT | 2019 | Effect of Foot Reflexology and Aromatherapy on Anxiety and Pain During Brachytherapy for Cervical Cancer | Multiple Interventions |
| Miller et al.[52] | RCT | 2021 | Pain and Spirituality Outcomes Among Women With Advanced Breast Cancer Participating in a Foot Reflexology Trial | Outcomes not relevant |
| Noh et al.[52] | RCT | 2019 | Effects of aroma self-foot reflexology on peripheral neuropathy, peripheral skin temperature, anxiety, and depression in gynaecologic cancer patients undergoing chemotherapy: A randomised controlled trial | Multiple Interventions |
| Rottman et al.[52] | RCT | 2020 | Expectancy and Utilisation of Reflexology among Women with Advanced Breast Cancer | Outcomes not relevant |
